# Comparative Study of Properties and Cytotoxicity of Eco-Friendly AgInS_2_, CuInS_2_, and ZnIn_2_S_4_ Quantum Dots under a Unified Experimental Framework

**DOI:** 10.1021/acsomega.6c09767

**Published:** 2026-10-21

**Authors:** Cecília B. da Silva, Letícia R. C. Cunha, Paulo H. A. Campos-Junior, Arnaldo C. Pereira, Dmitry Aldakov, Marco A. Schiavon

**Affiliations:** † Materials Chemistry Research Group, Department of Natural Sciences, Federal University of São João del-Rei, Dom Bosco Campus, São João del-Rei, MG 36301-160, Brazil; ‡ Laboratory for Biology of Fertility Research, Department of Natural Sciences, Federal University of São João del-Rei, Dom Bosco Campus, São João del-Rei, MG 36301-160, Brazil; § Polymer and Electroanalytics Research Group, Department of Natural Sciences, Federal University of São João del-Rei, Dom Bosco Campus, São João del-Rei, MG 36301-160, Brazil; ∥ Université Grenoble Alpes, CNRS, CEA, IRIG, SYMMES, STEP, 38000 Grenoble, France

## Abstract

Ternary quantum dots (TQDs) are promising cadmium-free nanomaterials for optoelectronic and biomedical applications; however, systematic comparisons among AgInS_2_ (AIS), CuInS_2_ (CIS), and ZnIn_2_S_4_ (ZIS) quantum dots remain limited because these materials are typically investigated using different synthetic protocols and characterization methodologies. Herein, we present a systematic comparative study of aqueously synthesized AIS, CIS, and ZIS quantum dots, enabling direct comparison of their optical, electrochemical, surface, and biological properties within a unified comparative framework. The nanocrystals exhibited similar sizes (4–6 nm), ensuring that the observed differences primarily reflected composition. Combined optical and electrochemical analyses revealed consistent trends in bandgap energies that were in good agreement with Mulliken electronegativity-based predictions. Despite their structural similarity, AIS showed long lifetimes and moderate emission efficiency, CIS exhibits suppressed photoluminescence due to dominant nonradiative recombination, whereas ZIS exhibited fast recombination dynamics associated with surface-dominated states. X-ray photoelectron spectroscopy established a direct relationship between composition, defect states, and electronic structure, while all QDs exhibited high cell viability (>90%), confirming their cytocompatibility. Overall, this unified comparative approach demonstrates that cation identity governs band alignment, recombination pathways, and cytocompatibility, providing practical guidance for the rational design of cadmium-free quantum dots for photocatalytic, optoelectronic, and biomedical applications.

## Graphical abstract

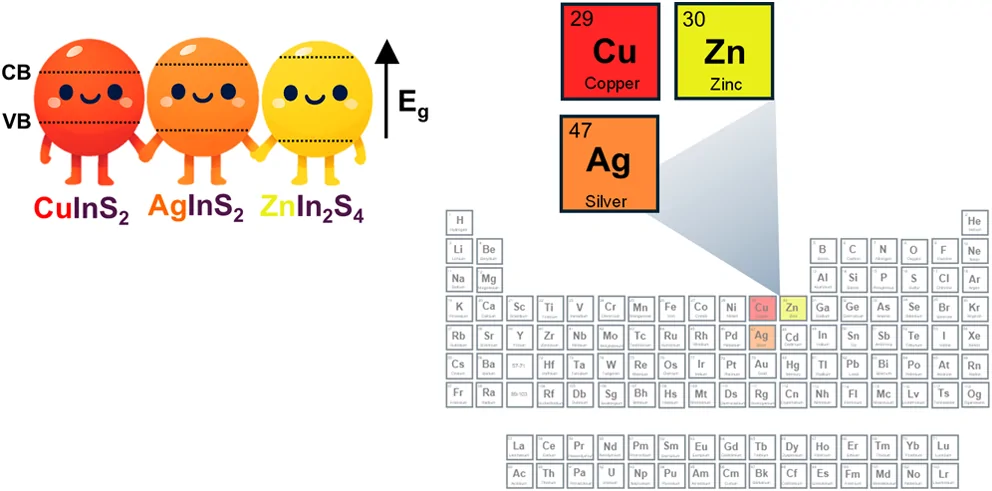

## 1 Introduction

Quantum dots (QDs) have been investigated for over four decades.^1^ Nevertheless, these colloidal semiconductor nanomaterials continue to attract considerable attention because their optoelectronic properties can be precisely tuned by controlling their size, composition, and surface chemistry size.^2^ This versatility has enabled their application in a broad range of technologies, including solar cells,^3^ light-emitting diodes (LEDs),^4^ photocatalysis,^5^ and bioimaging,^6^ among many others.

Binary QDs from the II–VI family (CdTe/CdSe) are widely described in the literature. However, the inherent toxicity of cadmium has limited their practical and biomedical applications, stimulating the development of environmentally benign alternatives. In this context, ternary quantum dots (TQDs), specifically those belonging to the I–III–VI (AgInS_2_ and CuInS_2_) and II–III–VI (ZnIn_2_S_4_) groups, have emerged as promising cadmium-free nanomaterials.^7^ TQDs exhibit direct bandgap, tunable band structure, strong absorption, band-edge or defect-related photoluminescence (PL).^8^ Unlike binary QDs, TQDs are typically synthesized under off-stoichiometric conditions, leading to the formation of intrinsic defects such as vacancies, and antisite substitutions. These defects promote radiative recombination through donor–acceptor pairs and surface-related states, giving rise to broad emission bands whose characteristics strongly depend on composition. Consequently, the combination of defect-mediated luminescence, compositional tunability, such as bioimaging, photocatalysis, and solar energy conversion.^9^

TQDs are commonly synthesized in organic media using high-boiling-point solvents, inert atmospheres, and strongly coordinating ligands, enabling precise control over crystal growth and high photoluminescence efficiencies. However, these methods are often associated with high cost, complex processing, and the use of hazardous reagents. Direct one-pot aqueous synthesis has therefore emerged as a simple, cost-effective, and environmentally friendly alternative, allowing the preparation of hydrophilic nanocrystals under mild conditions without postsynthetic ligand exchange. Nevertheless, aqueous routes frequently produce broader size distributions and higher defect densities than organic syntheses, significantly affecting the optical and electronic properties of the resulting QDs.^10^ Understanding how aqueous synthesis influences the structure, defect landscape, and electronic behavior of TQDs is therefore essential for expanding their practical applications.

The electronic structure of TQDs can be manipulated by adjusting two main parameters: size and composition. According to theoretical calculations, the elements from group III (In and Ga) are identified to contribute to the conduction band (CB), whereas those from group I (Cu and Ag) to the valence band (VB). Consequently, changes in cation composition directly influence both the bandgap energy (*E*_g_) and the positions of band edges.^11,12^

Among I–III–VI ternary semiconductors, silver indium disulfide QDs, AgInS_2_ (AIS), have attracted considerable attention because of their high ionic mobility and efficient cation exchange, which can occur even at relatively low synthesis temperatures. These characteristics promote extensive defect formation and broad compositional tunability. AIS exhibits a direct bandgap ranging from 1.54 to 2.50 eV, depending on the crystal structure.^13^ Compared with other TQDs, AIS QDs offer favorable chemical stability because it does not contain cations with multiple oxidation states, such as Cu^2+^/Cu^+^. The strong Lewis acid–base interaction between Ag^+^ and S^2–^ facilitates the rapid formation of stable Ag–S bonds during synthesis. Nevertheless, partial substitution of Ag^+^ by In^3+^ commonly occurs, leading to off-stoichiometric compositions and the formation of intrinsic and surface defects, including vacancies, antisites, and dangling bonds. These defect states strongly influence the optical response of AIS by introducing additional radiative recombination pathways.^14^ The VB of AIS QDs is mainly composed of S 3p orbitals hybridized with Ag 4d orbitals, whereas the conduction band is formed by hybrid In 5p5s and S 3p orbitals.^15^

CuInS_2_ (CIS) QDs constitute another important class of I–III–VI ternary semiconductors owing to their broad visible-light absorption, large Stokes shift, a long PL lifetime, a tunable emission wavelength, and a direct bandgap of 1.45–2.20 eV.^5^ Similar to AIS, CIS commonly exhibits intrinsic defects arising from copper and indium vacancies as well as antisite substitutions, which play a central role in determining its optical and electronic properties. Consequently, variations in stoichiometry provide an effective strategy for tailoring its photoluminescence and photoelectronic behavior. CIS QDs have been reported in three crystalline structures: tetragonal chalcopyrite, cubic, and hexagonal wurtzite.^16^ The electronic band structure of CIS QDs comprises a VB of Cu 3d and S 3p orbitals, while the CB comprises the antibonding states Cu 4s, S 3p, and In 5s.^17^

In contrast to AIS and CIS, ZnIn_2_S_4_ (ZIS) belongs to the II–III–VI semiconductor family and is the only member of the AB_2_X_4_ ternary chalcogenide system. ZIS is an n-type semiconductor with a direct bandgap (2.02–2.85 eV), high chemical stability, and a large absorption coefficient. ZIS typically exhibits two crystal structures: cubic sphalerite (zinc blende) and hexagonal wurtzite.^18^ The VB of ZIS QDs is mainly composed of Zn 3d, In 5s/5p, and S 3p orbitals, whereas the CB is primarily formed by In 5s/5p and S 3p states, with a minor contribution from Zn 3d.^19^ Although numerous ZIS nanostructures, including microspheres, nanoflowers, nanosheets, nanotubes, and nanoribbons,^20^ the synthesis of zero-dimensional ZIS QDs with uniform size remains challenging due to their intrinsic layered structure. Consequently, reports on well-defined ZIS QDs are still relatively limited.^21–23^

Although AIS, CIS, and ZIS QDs have been extensively investigated individually, direct comparisons among these materials remain relatively scarce because they are typically synthesized using different protocols and subsequently characterized under different experimental conditions. Consequently, distinguishing intrinsic composition-dependent effects from variations introduced by synthetic and analytical methodologies remains challenging. Since the dominant cation (Ag^+^, Cu^+^, or Zn^2+^) plays a fundamental role in determining the electronic structure, defect chemistry, and recombination pathways of ternary quantum dots, establishing a unified comparative framework is essential to elucidate how cation identity governs their structural, optical, electrochemical, surface, and biological properties.

In this work, we present a systematic comparative study of AIS, CIS, and ZIS QDs synthesized *via* aqueous one-pot routes under comparable, but composition-specific, conditions, with specific adjustments made for ZIS QDs due to their distinct formation behavior. These three QD systems were selected to enable a direct assessment of the effects of the dominant cation (Ag^+^, Cu^+^, or Zn^2+^) on their physicochemical and biological properties. Rather than proposing a new synthetic methodology, the novelty of this study lies in the systematic comparison of these three compositions using complementary optical, structural, electrochemical, surface, and biological characterization under a common comparative strategy. This integrated approach enables direct composition/property correlations and provides insights into how cation identity influences band alignment, defect states, recombination pathways, and cytocompatibility in cadmium-free QDs.

## 2 Experimental Section

### 2.1 Materials

Copper­(II) chloride dihydrate (CuCl_2_·2H_2_O, 99.0%), indium chloride (InCl_3_·4H_2_O, 99.7%), sodium sulfide (Na_2_S·9H_2_O, 98.0%), zinc acetate (Zn­(OAc)_2_, 99.99%), sodium hydroxide (NaOH, 97.0%), 3-mercaptopropionic acid (MPA, 99.0%), tetrabutylammonium hexafluorophosphate (TBAPF_6_, 98.0%), glucose, and 3-[4,5-dimethylthiazol-2-yl]-2,5-diphenyl tetrazolium bromide (MTT) were purchased from Sigma-Aldrich. Silver nitrate (AgNO_3_, P.A.) was obtained from Cennabras. Ammonium hydroxide (NH_4_OH, P.A.) and thioglycolic acid (TGA, 99.0%) were purchased from Synth. Anhydrous sodium sulfate (Na_2_SO_4_, 99.0%), ethyl alcohol (99.8%), and acetonitrile (99.8%) were purchased from Vetec. Colorless gelatin powder (24.0 g) is commercially available. Fetal bovine serum, Dulbecco’s Modified Eagle Medium (DMEM), penicillin, and streptomycin were purchased from Gibco. All chemicals were used without further purification. Ultrapure water was used throughout the experiments.

### 2.2 Synthesis of the AIS and CIS QDs

The synthesis of AIS and CIS QDs was adapted from the aqueous method reported by ref 24, with modifications to the experimental procedure as described below. AIS QDs were prepared by adding 20 μL of silver nitrate (1.0 mol L^–1^) and 100 μL of indium chloride (1.0 mol L^–1^) to 10.0 mL of ultrapure water in a flask. Subsequently, 800 μL of MPA (1.0 mol L^–1^) was added, and the solution pH was adjusted to 10 with 25% aq. ammonium hydroxide. Subsequently, 200 μL of sodium sulfide (1.0 mol L^–1^) was added at room temperature. The solution was heated in a water bath at 100 °C for 120 min under stirring. For the synthesis of CIS QDs, the silver precursor was replaced by 20 μL of copper chloride (1.0 mol L^–1^). After synthesis, the QDs were purified by precipitation with acetone, followed by centrifugation at 6080*g* for 10 min. The precipitate was then redispersed in ultrapure water before characterization. The schematic models for the synthesis of AIS and CIS QDs are shown in Figure S1a,b, respectively.

### 2.3 Synthesis of the ZIS QDs

The synthesis of ZIS QDs was adapted from the aqueous synthetic protocol reported in,^21^ with modifications to the experimental procedure, as described below. ZIS QDs were prepared by adding 0.063 mmol of zinc acetate and 0.25 mmol of indium chloride to a flask containing 20.0 mL water. Then, 0.15 g of gelatin and 20.0 mL of ultrapure water at 80 °C were added simultaneously to the solution, and the mixture was stirred until complete dissolution. Gelatin was introduced as an additional macromolecular stabilizing agent to control the growth and aggregation of ZIS nanostructures, favoring the formation of zero-dimensional QDs and improving their colloidal stability. Its use was particularly relevant for ZIS because of the intrinsic tendency of this composition to form lamellar structures. Subsequently, 1.5 mL of TGA was added to the solution, and the pH was adjusted to 7 with 2 mol L^–1^ sodium hydroxide solution. Then, 1.25 mmol of sodium sulfide was added. The solution was heated in an oil bath at 150 °C for 180 min under vigorous stirring. Figure S1c shows the schematic model for the synthesis of ZIS QDs. Following synthesis, the ZIS QDs were isolated by acetone precipitation, centrifuged at 6080*g* for 10 min, and finally redispersed in ultrapure water before characterization.

### 2.4 Characterization of the TQDs

Absorption spectra were recorded using a UV-2550 SHIMADZU diode array spectrophotometer in the 325–825 nm range, using water as the solvent and quartz cells with a 1 cm optical path (Hellma). Emission spectra were collected on an RF-5301PC SHIMADZU spectrofluorometer using a 150 W xenon lamp as the light source. Samples were measured in quartz cuvettes (1 cm path length) over the 425–900 nm range. Excitation wavelength was 450 nm for AIS and CIS, and 380 nm for ZIS QDs. The excitation wavelength was selected to maximize the PL intensity, since the emission maximum remained essentially unchanged with different excitation wavelengths. Photoluminescence quantum yields (PLQY) were determined using the comparative method described by Williams et al.^25^ by comparing the integrated emission of the QDs with reference standards. Rhodamine 101 solution (PLQY: 95% in ethyl alcohol, with emission peak of 608 nm) was used for AIS and CIS QDs, and quinine sulfate solution (PLQY: 54% in water, with emission peak of 450 nm) was used for ZIS QDs. Time-resolved photoluminescence (τ) was obtained using a Horiba Fluorolog-3 Jobin-Yvon spectrofluorometer with excitation using a 455 nm pulsed nanoLED. The τ curves were fitted using a biexponential function, provided in the Supporting Information (eq S1). For graphical comparison, the experimental decay curves shown in Figure 1d were normalized by their maximum intensity (*I*/*I*_0_) after data export. The crystalline structure of the materials was analyzed by X-ray diffraction (XRD) using a Shimadzu diffractometer with radiation, Kα of Cu, λ = 1.5418 Å, graphite monochromator and sweep of 0.02°, in the (10–80° 2θ range). The infrared spectra were obtained on a Bruker Fourier transform infrared spectrometer (ATR) between 4000 and 400 cm^–1^, with a resolution of 4 cm^–1^. Transmission electron microscopy (TEM) images were obtained using a FEI Tecnai G2–20 SuperTwin operated at 200 kV. The samples were prepared by drop-casting onto a 400-mesh carbon grid covered with an ultrathin carbon film (∼3 nm). Data analysis was performed using ImageJ. X-ray photoelectron spectroscopy (XPS) analyses were performed using a Versa Probe II spectrometer (ULVAC-PHI) equipped with a monochromated Al Kα source (*h*ν = 1486.6 eV), using purified QD powders obtained after the synthesis procedure. The core-level peaks were recorded at a constant pass energy of 23.3 eV. The XPS spectra were fitted using the CasaXPS 2.3 software with Shirley background. Binding energies were referenced with respect to adventitious carbon (C 1s BE = 284.8 eV).

**Figure 1:**
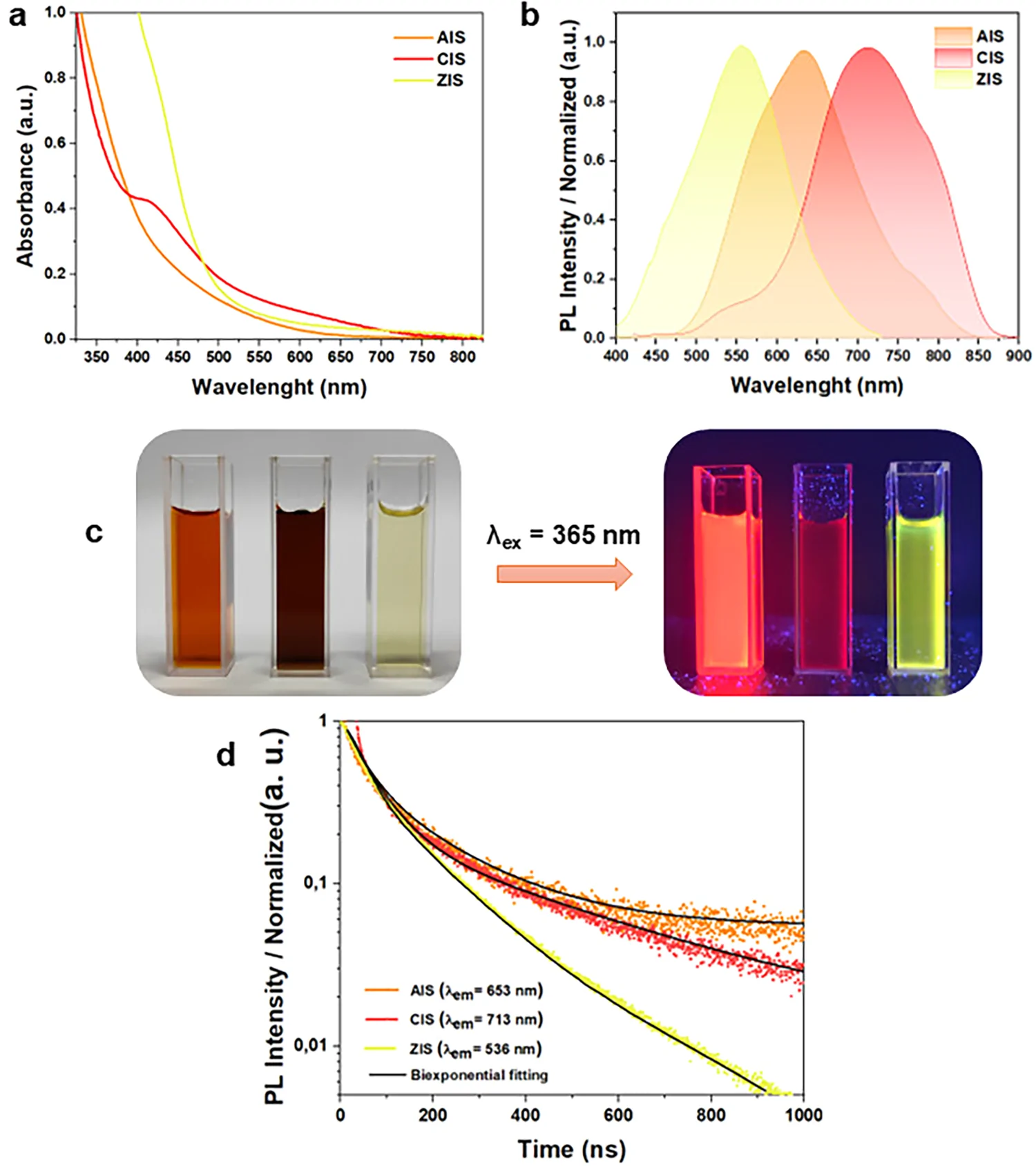
(a) UV–vis absorption, (b) normalized PL spectra, and (d) PL decay curves of AIS, CIS, and ZIS QDs and their corresponding biexponential fits; (c) from left to right: solutions of AIS, CIS, and ZIS QDs under ambient and UV light.

All electrochemical measurements were performed using a PGSTAT204 Metron Autolab potentiostat with NOVA 2.1.6 software. The experimental setup for electrochemical measurements consisted of three electrodes inserted in a homemade Teflon 3-neck. For impedance spectroscopy and Mott–Schottky analysis, fluorine-doped tin oxide (FTO) glass substrates coated with TQDs were used as working electrodes, with 0.1 mol L^–1^ Na_2_SO_4_ as the supporting electrolyte. In contrast, cyclic voltammetry (CV) experiments were conducted using a glassy carbon electrode (GCE) modified with TQDs as the working electrode, and 0.1 mol L^–1^ tetrabutylammonium hexafluorophosphate (TBAPF_6_) in acetonitrile as the supporting electrolyte. In both cases, an Ag/AgCl (3.0 mol L^–1^ KCl) electrode and a platinum wire were used as the reference and counter electrodes, respectively. The working electrodes were prepared by dispersing 15.0 mg of the TQDs in 1 mL of absolute ethanol and 100 μL of Nafion solution. The mixture was sonicated for 1 h to obtain homogeneous dispersion. Subsequently, 5 μL of the suspension was drop-cast onto the conductive side of the substrate (FTO or glassy carbon), covering an area of approximately 1 cm^2^ (in the case of the FTO substrate), and dried under an infrared lamp for 10 min to remove volatile organic compounds before use. To ensure the reliability of the electrochemical measurements, the deposition of the QDs in Nafion was carried out using a fixed solution volume, controlled concentration, and standardized drying conditions. The electrochemical signals obtained showed good consistency among measurements performed under the same conditions, indicating that the drop-cast film was suitable for the analysis. In addition, the use of Nafion provided strong adhesion of the QDs to the substrate, preventing detachment during the scans and ensuring that the results reflected the intrinsic behavior of the nanocrystals synthesized in aqueous medium. The CV curves were obtained at a scan rate of 30 mV s^–1^ in the potential range of −2.0 to 2.0 V, whereas the Mott–Schottky measurements were performed in the applied potential range between −1 and 1 V, with a scan frequency of 10 kHz. All measurements were performed at room temperature.

### 2.5 Cytotoxicity Assay of the TQDs

The cytotoxicity assay of TQDs against Chinese hamster ovary epithelial cells (CHO) was evaluated using the MTT assay. CHO cells were cultured in DMEM containing 4.5 mg L^–1^ glucose supplemented with 10% fetal bovine serum and 1% antibiotic (100 IU mL^–1^ penicillin, 100 μg mL^–1^ streptomycin) until the third passage in an incubator with a humidified atmosphere and 5% CO_2_ at 37 °C. The QDs were weighed and sterilized by UV exposure for 20 min. They were then mixed with the culture medium, which was supplemented to achieve a stock concentration of 1 mg mL^–1^. The tested concentrations (150, 80, and 20 μg mL^–1^) were prepared from the stock solution. The MTT assay was performed by placing 1.0 × 10^4^ CHO cells/well in 96-well plates. After 24 h in contact with QDs at concentrations of 20.0 μg mL^–1^, 80.0 μg mL^–1^, and 150.0 μg mL^–1^ for 24 h, 100 μL of the culture medium was removed, and 10 μL of 10% (v/v) MTT solution was added for 4 h. Subsequently, 100 μL of sodium dodecyl sulfate (SDS) was added to dilute the synthesized Fformazan for spectrophotometry at 570 nm. For each experimental condition, six wells were analyzed, corresponding to two technical triplicates, and the experiment was performed once (one independent experiment). Before statistical comparison, the data were assessed for normality. The D’Agostino–Pearson test was not considered appropriate because of the limited sample size in some experimental groups. Therefore, normality was primarily assessed using the Shapiro–Wilk test, which indicated a normal distribution for all groups analyzed (*p* > 0.05). Additionally, the Kolmogorov–Smirnov test corroborated these results for the groups with sufficient sample size. Based on these analyses, differences among the Control, 20, 80, and 150 μg mL^–1^ groups were evaluated using one-way ANOVA followed by Tukey’s multiple-comparison test, with statistical significance set at *p* < 0.05.

## 3 Results and Discussion

The optical, electronic, surface, and biological properties of AIS, CIS, and ZIS QDs were systematically investigated to establish correlations between composition and functional behavior. The nanocrystals were synthesized using established aqueous one-pot protocols adapted and optimized for each composition, followed by systematic characterization within a unified experimental framework that enabled direct comparison of their optical, electrochemical, surface, and biological properties. Although all three QDs were synthesized under aqueous conditions, the protocols were adjusted according to the specific chemistry of each composition. While thiol-based ligands were sufficient to stabilize AIS and CIS QDs, ZIS QDs exhibited an intrinsic tendency to form lamellar structures. Therefore, gelatin was introduced as an additional stabilizing agent to promote the formation of 0D nanostructures and ensure colloidal stability. This comparative framework provides a consistent basis for correlating composition-dependent variations in defect chemistry and electronic structure with the functional properties of these environmentally friendly TQDs.

### 3.1 Optical Properties of the TQDs

To explore the influence of composition on the optical response of the synthesized QDs, their UV–visible absorption properties were first investigated. The UV–visible absorption spectra of AIS, CIS, and ZIS QDs are displayed in Figure 1a. The absorption spectra of AIS and ZIS QDs exhibit featureless absorption profiles with a gradual tail extending toward lower energies, a characteristic commonly attributed to compositional heterogeneity, size distribution, and the presence of defect states in the nanoparticles.^26,27^ In contrast, CIS QDs display a weak absorption shoulder at *ca*. 420 nm, which has been associated with the intrinsic electronic structure of I–III–VI_2_ TQDs, where compositional disorder, off-stoichiometric growth, and defect-related states contribute to the appearance of broad and structured absorption features.^28^ Although well-defined excitonic peaks are absent in all samples, the absorption onset of ZIS QDs is shifted toward higher energies, indicating a larger bandgap compared to AIS and CIS QDs. This behavior is associated with the distinct electronic structure of ZIS, where the valence band derives from hybridized Zn 3d, In 5s/5p, and S 3p orbitals, while the conduction band is mainly composed of In 5s/5p and S 3p states with minor Zn 3d contributions.^19^ Compared with Cu^+^ and Ag^+^, the fully occupied Zn^2+^ 3d^10^ orbitals exhibit weaker p–d repulsion with S 3p states, leading to a deeper valence band and consequently to a wider bandgap and blue-shifted absorption onset. In contrast, the incorporation of Ag in place of Cu strongly affects the VB energy, modifies the relative position of the cation *d*-levels, and alters the extent of *p-d* hybridization.^29^

Following the UV–visible absorption analysis, the photoluminescence properties of the synthesized QDs were investigated to evaluate how composition influences their radiative recombination processes. The PL spectra show peaks at 536, 653, and 713 nm for ZIS, AIS, and CIS QDs, respectively (Figure 1b). The broad peaks are characteristic of I–III–VI_2_ TQDs and have been attributed to mechanisms such as radiative recombination involving donor–acceptor pair (DAP), recombination of a hole with an electron from the conduction band, exciton self-confinement, or a combination of these factors.^30^ Typically, DAP recombination is the most recognized emission mechanism for TQDs. For AIS QDs sulfur vacancies and silver interstitials have been proposed as donor states, whereas silver vacancies and sulfur interstitials act as acceptor states in AIS QDs.^31^ Similarly, for CIS QDs, sulfur vacancies are commonly associated with donors and copper vacancies as acceptors states. In addition, the PL spectra of the AIS and CIS QDs showed lower-intensity bands at 600 and 550 nm, respectively, possibly attributable to residual In_2_S_3_ or ultrasmall AIS and CIS QDs.^32^ Although the emission mechanism of ZIS QDs remains less established, its emission is generally attributed to defect-related recombination processes, involving donor states associated with sulfur sites and acceptor states related to zinc vacancies.^33^ These observations indicate that, although the nature of the defect states varies with composition, defect-related radiative recombination remains the dominant emission mechanism in all three environmentally friendly TQDs.

To evaluate the thermal stability of the optical properties of the synthesized QDs, the temperature dependence of their UV–vis absorption and photoluminescence spectra was investigated over the range of 5–60 °C (Figures S2). The absorption spectra of the TQDs exhibited negligible changes with increasing temperature. In contrast, the PL intensity gradually decreased with increasing temperature, most likely due to thermal quenching arising from the activation of nonradiative surface trap states.^34^ Among the three materials, CIS QDs exhibited a noticeable red shift in the PL emission maximum, particularly above 20 °C. In this case, thermal energy prevents exciton localization as temperature increases, due to the cumulative effect of lattice expansion and electron–phonon interaction.^35^

The normalized time-resolved photoluminescence (τ) decay curves of AIS, CIS, and ZIS QDs are shown in Figure 1d, while the lifetime values were obtained by biexponential fitting, as described in the Supporting Information. The decay components indicate the presence of multiple recombination pathways, where the shorter lifetime is often attributed to band-edge recombination, while the longer lifetime is generally associated with defect-induced radiative processes.^36^ The average PL lifetimes (eq S2) of the AIS, CIS, and ZIS QDs were calculated (Table 1).

**Table 1:** Fit Parameters for Decay Times and PL Quantum Yields, and Radiative and Non-Radiative Decay Rates of TQDs^a^

sample	*A*_1_	τ_1_ (ns)	*A*_2_	τ_2_ (ns)	τ_average_ (ns)	PLQY (%)	*k*_r_ (s^–1^)	*k*_nr_ (s^–1^)
AIS	21.9	159.87 ± 7.95	78.1	657.33 ± 7.25	625.6	13.6	2.17 × 10^5^	1.38 × 10^6^
CIS	26.6	56.60 ± 1.07	73.3	358.48 ± 2.32	342.1	2.7	7.89 × 10^4^	2.84 × 10^6^
ZIS	53.3	1.75 ± 0.03	46.7	6.63 ± 0.05	5.5	9.9	1.80 × 10^7^	1.64 × 10^8^

a *A*_1_ and *A*_2_ are the amplitudes of the biexponential decay components; τ_1_ and τ_2_ are the corresponding decay lifetimes; τ_avarage_ is the average photoluminescence lifetime; PLQY is the photoluminescence quantum yield; *k*_r_ and *k*_nr_ are the radiative and nonradiative decay rate constants, respectively.

The longest decay time was observed for AIS QDs (625.6 ns), followed by CIS QDs (342.1 ns) and ZIS QDs (5.5 ns). For AIS and CIS QDs, long PL decay lifetimes observed are typical for TQDs due to their structural complexity, trap states, and surface defects.^37^ In contrast, ZIS QDs exhibit significantly faster recombination dynamics, which is much less studied as mentioned above. Previous studies on ZIS QDs have reported rapid carrier recombination and limited charge separation.^38,39^ This behavior may be related to differences in surface states, defect distribution, or recombination pathways, although a detailed mechanism cannot be conclusively established based on the present data. Within the unified comparative framework employed in this work, these results highlight the distinct recombination dynamics of the three compositions.

To further evaluate the excited-state relaxation pathways of the TQDs, the radiative (*k*_r_) and nonradiative (*k*_nr_) decay rate constants were calculated from the PLQY and average PL lifetime values (Table 1). AIS exhibited a higher radiative contribution and lower nonradiative decay rate than CIS, consistent with its higher PLQY. In contrast, CIS showed a lower radiative contribution and a higher nonradiative decay rate, which is consistent with its lower emission efficiency. ZIS exhibited substantially faster radiative and nonradiative decay processes, as reflected by its markedly shorter average lifetime. Despite the predominance of nonradiative relaxation, its radiative decay rate remained higher than those of AIS and CIS, consistent with the intermediate PLQY observed for ZIS. Overall, these results indicate that the cation composition influences the balance between radiative and nonradiative relaxation pathways and contributes to the distinct photoluminescence behavior of the TQDs.

The PLQY was determined to assess the emission efficiency of the synthesized QDs. The PLQY values for the AIS, CIS, and ZIS QDs were 13.6, 2.7, and 9.9%, respectively Table 1, with the calculation procedure described in the Supporting Information. Among the investigated TQDs, AIS exhibited the highest PLQY, followed by ZIS, whereas CIS presented the lowest value, highlighting the pronounced influence of composition on the emission efficiency of these TQDs. These values are within the typical range reported for core-only TQDs, which generally exhibit low to moderate PLQY limited by intrinsic defects and surface trap states. Strategies such as the insertion of a ZnS shell can be employed to improve PLQY; however, this approach is beyond the scope of the present work.^40^

To further investigate the electronic structure of the synthesized TQDs, their optical bandgap energies were initially estimated using the Tauc plot method. Since all three materials are expected to exhibit a direct bandgap, the Tauc plot was constructed according to the procedure described in the Supporting Information. The resulting bandgap values are summarized in Table 2, while the corresponding Tauc plots are presented in Figure S3a–c. This method assumes that the material exhibits negligible absorption below the bandgap energy; however, this assumption may not hold for doped semiconductors, those with structural defects as they can introduce intraband gap states, resulting in the appearance of Urbach tails in the absorption spectrum, which compromises the accuracy of the Tauc method.^41^

**Table 2:** Optical Properties and Bandgap Estimation of TQDs Using the Tauc Plot and 2nd Derivative Methods

sample	emission λ_max_ (nm)	*E*_g_ from Tauc plot (eV)	*E*_g_ from the 2nd derivative of absorption (eV)
AIS	653	2.71	2.20
CIS	713	2.51	1.96
ZIS	536	2.78	2.42

To overcome the limitations of the Tauc method in spectra without well-defined features, the absorption edge was additionally estimated using the second derivative of the absorption spectrum (d^2^ *A*/dλ^2^)^42,43^ (Figure S3d–f). Although it enhances weak inflection points and improves edge detection in featureless spectra, its accuracy decreases when the absorption profile lacks identifiable transitions. *E*_g_ values obtained from second derivative absorption for AIS, CIS, and ZIS QDs were 2.20, 1.96, and 2.42 eV, respectively (Table 2 and Figure S3a–f). The higher bandgap observed for ZIS QDs is consistent with the intrinsic electronic structure of ZIS, which naturally exhibits a wider separation between the valence and conduction bands compared with Ag- or Cu-based I–III–VI semiconductors.^44^ AIS and CIS QDs exhibit strong *p-d* hybridization involving Ag-4d/S-3p or Cu-3d/S-3p orbitals, which elevates the VB maximum and results in narrower bandgaps.^45^ Table S1 summarizes the optical bandgap values of AIS, CIS, and ZIS QDs reported in the literature using different synthesis routes and estimation methods, alongside the values obtained in this work. The comparison highlights the broad range of reported bandgap energies, which reflects the strong influence of factors such as particle size, composition, defect states, and synthesis conditions on the optical properties of TQDs.

### 3.2 Electronic Properties of the TQDs

Following the optical characterization, the electronic properties of the synthesized TQDs were investigated to establish their band-edge positions and electronic structure. The absolute CB and VB positions of semiconductors are first estimated using the Mulliken electronegativity approach. This method relates the band edge positions to the absolute electronegativity (χ) of the semiconductor, defined as the arithmetic mean of the electron affinity and the first ionization potentials of the constituent atoms.^46,47^ According to this model, the VB and CB can be calculated using, respectively, eqs 1 and 2

1 $$E_{VB}=\chi +0.5E_{g}-E_{e}$$

2 $$E_{CB}=E_{VB}-E_{g}$$

where χ is the absolute electronegativity of the TQDs, *E*_e_ is the energy of free electrons on the hydrogen scale (typically 4.5 eV), and *E*_g_ is the optical bandgap calculated using the second absorption derivative method. The tabulated ionization potential (IP) and electron affinity (EA) values used to determine χ were obtained from standard tabulated atomic data. Details of the Mulliken electronegativity calculations, including the calculation procedure, the equations employed, the stoichiometric weighting approach, and representative calculation examples for AIS, CIS, and ZIS QDs, are provided in the Supporting Information. The calculated values (Table 3) provide an approximate description of the electronic structure and serve as qualitative descriptors for comparison with the electrochemical measurements.

**Table 3:** Atomic Parameters, Mulliken Electronegativity, and Estimated Band-Edge Positions (*E*_VB_ and *E*_CB_) for AIS, CIS, and ZIS QDs^a^

sample	constituent atoms	NA	IP (eV)	EA (eV)	χ_atom_ (eV)	χ_sample_ (eV)	*E*_VB_ (eV)	*E*_CB_ (eV)
AIS	Ag	1	7.576	1.304	4.44	4.98	1.58	–0.62
	In	1	5.786	0.383	3.08			
	S	2	10.360	2.077	6.21			
CIS	Cu	1	7.725	1.236	4.48	4.99	1.47	–0.49
	In	1	5.786	0.383	3.08			
	S	2	10.360	2.077	6.21			
ZIS	Zn	1	9.393	–0.600	4.39	5.05	1.76	–0.66
	In	2	5.786	0.383	3.08			
	S	4	10.360	2.077	6.21			

a NA = stoichiometric coefficient of each constituent element; IP = ionization potential; EA = electron affinity; χ_atom_ = atomic electronegativity; χ_sample_ = sample electronegativity (Mulliken); *E*_VB_ = valence band edge potential; *E*_CB_ = conduction band edge potential.

Although the Mulliken electronegativity approach simplifies the electronic structure of the material, it provides theoretical estimates of the conduction- and valence-band positions that can be used as qualitative descriptors of the electronic structure. These theoretical estimates were considered together with the experimental results obtained from cyclic voltammetry and Mott–Schottky analysis to evaluate the relative energy-level alignment among AIS, CIS, and ZIS QDs.

Cyclic voltammetry (CV) was employed as an experimental approach to determine the *E*_CB_ and *E*_VB_ levels of the TQDs from their oxidation and reduction processes. Different criteria can be used to extract redox potentials from CV curves; here, onset potentials, rather than peak ones, were adopted as a more reliable descriptor of band-edge positions. The onset of oxidation corresponds to the energy level at which electron removal begins and is associated with the *E*_VB_, whereas the onset of reduction corresponds to the energy level at which electrons are accepted and is associated with the *E*_CB_. These onset values are preferred because they more accurately reflect the energetic limits of the band edges minimizing contributions from kinetic and capacitive effects present at peak potentials.^48^ For both oxidation and reduction, the selected values were determined from the first sustained deviation of the baseline corresponding to the main faradaic process. Minor current features preceding this transition were not considered because they are commonly attributed to surface- or defect-related electrochemical processes rather than to the intrinsic band-edge transitions of the TQDs.

Figure 2a–c shows the cyclic voltammograms for the AIS, CIS, and ZIS QDs in relation to the Ag/AgCl potential scale. The onset potentials (*E*_onset,ox._ and *E*_onset,red._) were identified with the intersection points of the tangents. The resulting *E*_CB_ and *E*_VB_ values were converted to the NHE scale and absolute vacuum scales, and the electrochemical bandgap was calculated from their difference (Table 4). The electrochemical bandgaps followed the order ZIS > AIS > CIS, consistent with the optical bandgap trend, highlighting the composition-dependent electronic structure of the TQDs. The values obtained are consistent with previously reported electrochemical band structures for similar TQDs systems (AIS and CIS QDs).^49,50^ Although ZnIn_2_S_4_ has been widely investigated for its optical and photocatalytic properties, experimental reports on the determination of band-edge positions of ZIS QDs by CV are still lacking. These results extend the comparative electrochemical characterization of AIS, CIS, and ZIS QDs investigated under the same experimental conditions.

**Figure 2:**
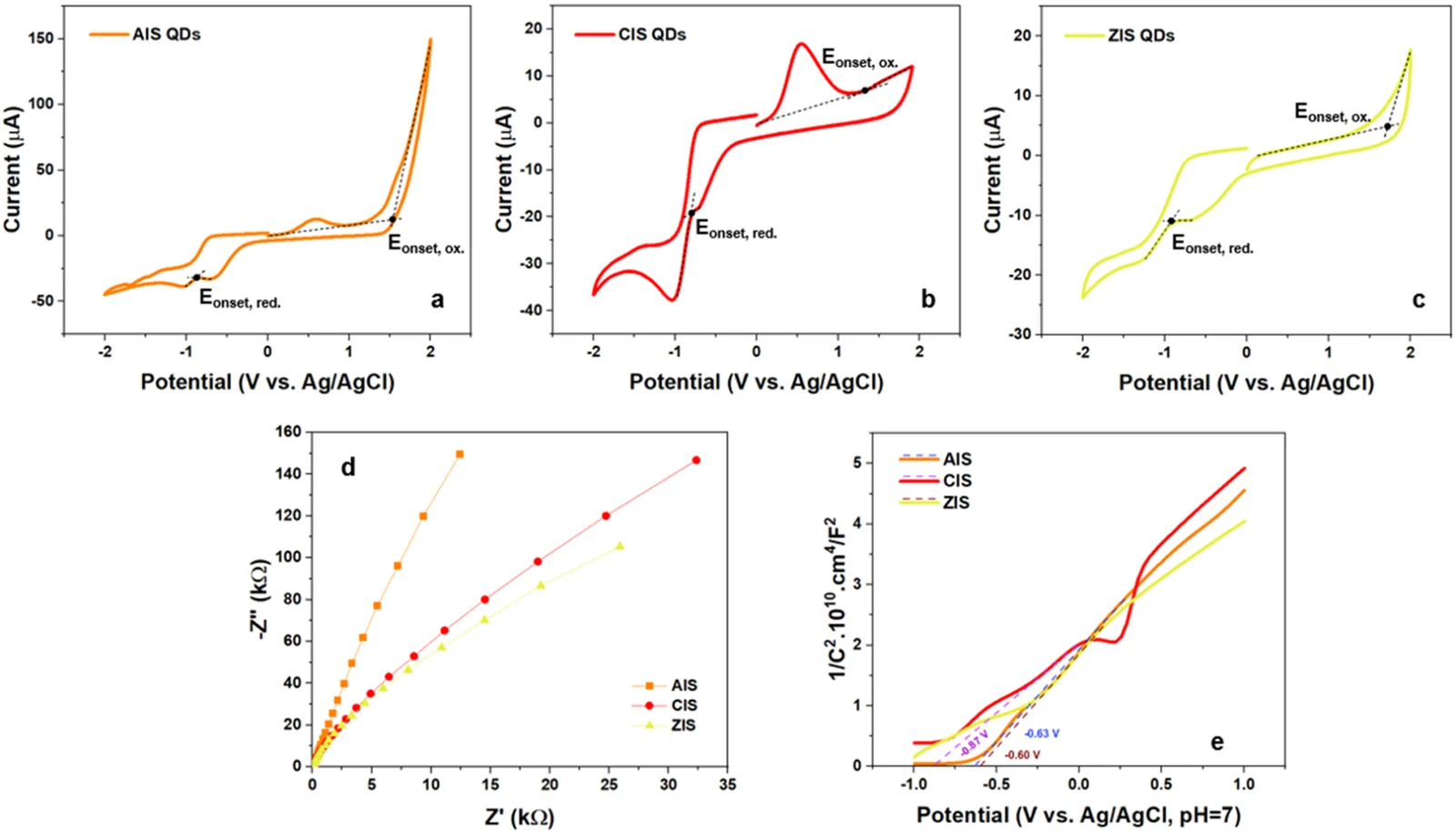
Electrochemical characterization of the TQDs. Cyclic voltammetry curves of (a) AIS, (b) CIS, and (c) ZIS QDs, showing the onset potentials of oxidation and reduction; (d) Nyquist plots; and (e) Mott–Schottky plots used to estimate the flat band potentials.

**Table 4:** Oxidation (*E*_onset,ox._) and Reduction Potential (*E*_onset,red._), VB, CB, and Electrochemical (*E*_c_) Bandgap Energies of TQDs

sample	*E*_onset,ox._ (V)	*E*_onset,red._ (V)	*E*_VB/NHE_ (eV)	*E*_CB/NHE_ (eV)	*E*_VB/AVS_ (eV)	*E*_CB/AVS_ (eV)	*E*_c_ (eV)
AIS	1.53	–0.88	1.73	–0.68	–6.23	–3.82	2.41
CIS	1.33	–0.81	1.53	–0.61	–6.03	–3.89	2.14
ZIS	1.72	–0.92	1.92	–0.72	–6.42	–3.78	2.64

Figure 2d presents the Nyquist plots obtained from electrochemical impedance spectroscopy (EIS) for TQDs films. The diameter of the semicircle at high frequencies is directly related to the charge transfer resistance (*R*_ct_), which reflects the ease of interfacial electron transport. Among the samples, the AIS QDs exhibit the largest semicircle, indicating a higher *R*_ct_ and slower charge transfer efficiency, whereas CIS and ZIS QDs show smaller semicircles, suggesting more efficient interfacial electron transport.^51^

Mott–Schottky (M–S) analysis was performed to further investigate the electronic structure of the semiconductor/electrolyte interface. This method enables determination of the semiconductor type (*n-* or *p-*) and the estimation of the flat-band potential (*V*_fb_), *E*_CB_, and *E*_VB_.^52^ Figure 2e displays the Mott–Schottky plots of AIS, CIS, and ZIS QDs films, recorded in 0.1 mol L^–1^ electrolyte at pH 7 (*vs* Ag/AgCl). All curves exhibit a positive slope, confirming the *n-*type semiconductor behavior of the samples.^52^

The *V*_0_ values were obtained from the *x*-axis intercepts of the linear regions of the Mott–Schottky plots. The resulting electrochemical parameters are provided in the Supporting Information. Then, *V*_fb_ was calculated

3 $$V_{0}=V_{fb}+\mathrm{RT}/F$$

*R* is the gas constant, *T* is the absolute temperature, and *F* is the Faraday.

The *E*_CB_ and *E*_VB_ were subsequently estimated as follows

4 $$E_{CB}=V_{fb}-\mathrm{kT}\ln{(N}_{C}/N)$$

5 $$E_{VB}=E_{CB}+E_{g}$$

Here, *k* is the Boltzmann constant, *N*_c_ is the effective density of states of the conduction band, and *N* is the carrier density. For *n*-type semiconductors, the *E*_CB_ is ∼0.1 V lower than its *V*_fb_ value.

The *E*_VB_ values were calculated using the optical bandgap (*E*_g_), obtained by the second absorption derivative (eq 5), and together with *E*_CB_ are summarized in Table S2. They reveal clear differences in band alignment among AIS, CIS, and ZIS QDs, which are expected to influence charge separation and interfacial processes.

The resulting band structures obtained from CV, M–S, and Mulliken approaches are presented in Figure 3a–c. The band-edge positions estimated by CV, M–S, and Mulliken approaches are presented in Figure 3a–c. Because these approaches are based on different physical principles, the absolute values obtained from them are not considered directly equivalent. Instead, the results are compared primarily in terms of the relative band-edge alignment and composition-dependent trends across the three systems.

**Figure 3:**
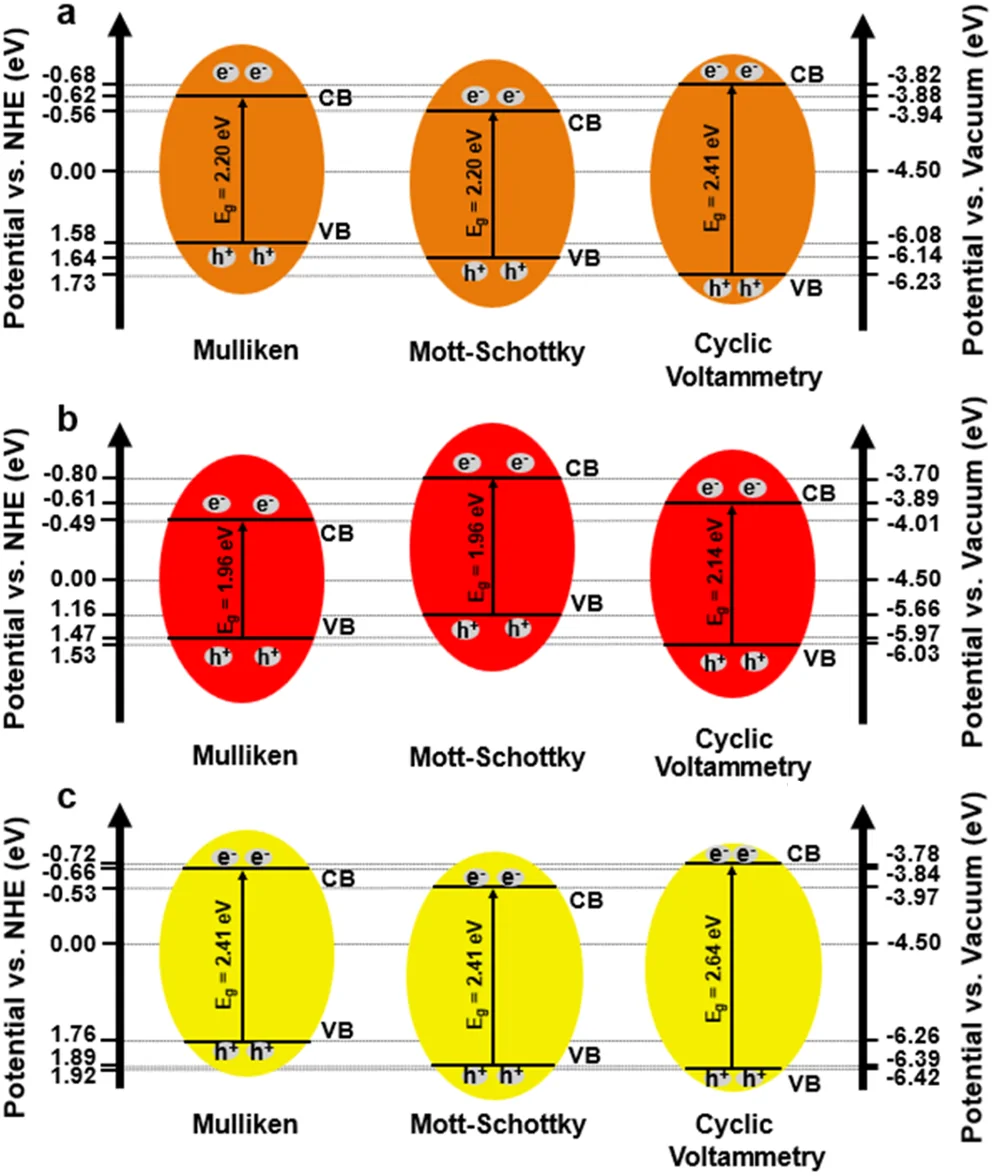
Energy band edge positions of (a) AIS, (b) CIS, and (c) ZIS QDs determined by Mulliken electronegativity calculations, Mott–Schottky analysis, and cyclic voltammetry. The CB and VB levels are referenced to both the NHE and the vacuum scale (AVS).

For AIS QDs (Figure 3a), the Mulliken calculations predict slightly more negative CB and VB edge positions than the experimental values, which are in close agreement, indicating a consistent band edge determination for this system. In the case of CIS QDs (Figure 3b), the M–S analysis yields higher band energy values compared to Mulliken and CV, suggesting a stronger influence of interfacial states on the extracted flat-band potential.^53^ For ZIS QDs (Figure 3c), the CB and VB positions are well aligned across all three approaches, with only minor variations, indicating a more uniform electronic structure determination. The higher VB of ZIS QDs ensures strong oxidative capability, whereas the more negative CB provides sufficient driving force for reduction reactions, highlighting its potential for photocatalytic applications such as water splitting.^54^

The electrochemical bandgap values obtained from the CV measurements were higher than the corresponding optical bandgap values. These differences are expected because the two approaches probe different aspects of the electronic structure and are influenced by different physical factors, including interfacial electrochemical processes and optical transitions involving excitonic and defect-related states.^49,55^

The differences observed among Mulliken, cyclic voltammetry (CV), and Mott–Schottky (M–S) methods are expected, as each approach probes the electronic structure based on distinct principles. The Mulliken method provides idealized theoretical estimates derived from the electronegativities of the constituent elements and assumes stoichiometric structures, whereas CV and M–S measurements reflect experimental conditions and interfacial phenomena. In colloidal AIS, CIS, and ZIS QDs, deviations from ideal stoichiometry, cation disorder, and intrinsic defects can lead to differences between theoretical and experimental values, making Mulliken-derived parameters more suitable as qualitative descriptors rather than exact quantities. In contrast, CV captures redox processes in the deposited films, whereas M–S analysis derives band-edge positions from capacitance responses at the semiconductor/electrolyte interface and is influenced by carrier density and film quality. Despite these differences, all methods consistently reproduce the same relative trends among AIS, CIS, and ZIS QDs, supporting the reliability of the proposed band alignment.

### 3.3 Chemical and Surface Characterization of the TQDs

To correlate the optical and electronic properties with the surface chemistry of the synthesized QDs, XPS analysis was performed to determine the elemental composition and surface electronic states. XPS analysis was performed to determine the elemental composition and surface electronic states of the as-prepared AIS, CIS, and ZIS QDs. Representative high-resolution spectra of the distinguishing metal species (Ag, Cu, and Zn) are presented in Figure 4, whereas the survey spectra, complete high-resolution spectra, elemental composition, and ligand-related spectra are provided in the Supporting Information.

**Figure 4:**
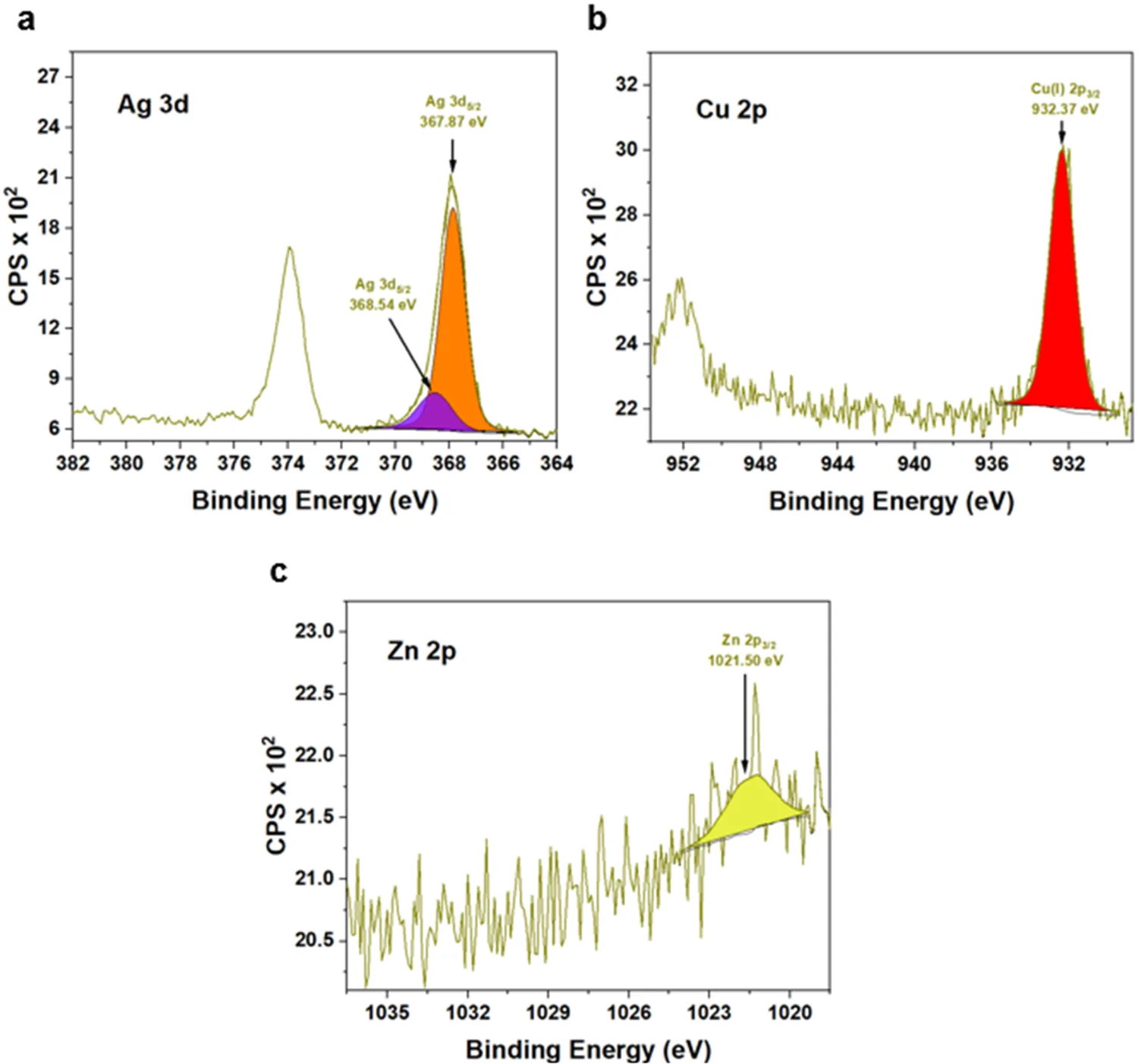
High-resolution XPS spectra of (a) Ag 3d in AIS QDs, (b) Cu 2p in CIS QDs, and (c) Zn 2p in ZIS QDs.

The survey spectra confirm the presence of the expected elements in each system, including In and S, which are common to all three materials, along with signals arising from surface ligands. The high-resolution In 3d spectra for AIS, CIS, and ZIS QDs exhibit the In 3d_5/2_ and In 3d_3/2_ peaks at ∼444.4–445.0 eV and ∼452.1–452.6 eV, consistent with In^3+^ in a sulfide environment across all systems. In AIS QDs, an additional component at ∼445.7 eV is attributed to defect-related sites (*e.g.*, In_Ag_ or V_Ag_) or surface effects,^27,56^ with similar features also present, although less pronounced, in CIS and ZIS QDs.

The S 2p spectra (Figure S5b,d,f) for AIS, CIS, and ZIS QDs exhibit the characteristic S 2p_3/2_ and S 2p_1/2_ peaks at ∼161.7–162.1 eV and ∼162.8–163.0 eV, respectively, corresponding to sulfide species (S^2–^) bound to metal atoms in the QDs lattice.^57,58^ In AIS and CIS QDs, an additional component at ∼163.5 eV is observed and attributed to sulfur from thiolate groups of the MPA ligand coordinated to Cu or In.^59^ In ZIS QDs, the main contribution at ∼163.4 eV is associated with sulfur from the TGA ligand, while the sulfide-related signal at ∼162.1 eV appears less intense. In all systems, weaker peaks at higher binding energies (∼168.0–170.0 eV) are assigned to oxidized sulfur species (SO_*x*_), likely originating from residual precursor- or ligand-related species (*e.g.*, Na_2_SO_4_).^60^ The relative intensity differences in the S 2p region reflect the surface-sensitive nature of XPS, which enhances the contribution of ligand-derived sulfur compared to the inorganic core.

The differences among the TQDs are mainly attributable to the Ag, Cu, and Zn components. For AIS QDs, the Ag 3d spectrum (Figure 4a) shows peaks at 367.87 and ∼373.80 eV, with a spin–orbit splitting of ∼6 eV, consistent with Ag^+^ in a sulfide environment.^61^ An additional component at 368.64 eV may originate from local variations in the chemical environment, such as antisite-related defects (In_Ag_)^62,63^ or, alternatively, from residual organic ligands and sample charging.

In CIS QDs, the Cu 2p spectrum (Figure 4b) exhibits peaks at 932.37 and 952.30 eV, corresponding to Cu^+^, with no satellite features, confirming the absence of Cu^2+^ species.^52^ For ZIS QDs, the Zn 2p spectrum (Figure 4c) shows a relatively weak feature centered at 1021.50 eV corresponding to Zn^2+^, consistent with its incorporation into a sulfide lattice.^39^

The compositional analysis (Table S3) reveals an Ag:In ratio of ∼1:4.5 for AIS QDs, which is consistent with the initial cation loading ratio used during synthesis (Ag:In = 1:5), indicating that the final composition reflects the Ag-poor conditions employed. In contrast, CIS QDs show a Cu:In ratio of ∼1:13.4. Although the initial cation loading ratio was also Cu:In = 1:5, these results indicate an In-rich/Cu-deficient surface composition relative to the nominal stoichiometry. This behavior is consistent with previous reports on CIS QDs, where Cu-deficient compositions are commonly observed and associated with the formation of copper vacancies and defect-rich chalcopyrite structures.^39,64^ For ZIS QDs, the Zn:In ratio determined by XPS (∼1:2) differs from the initial precursor loading ratio used in the synthesis (Zn:In = 1:4) but is closer to the expected ZnIn_2_S_4_ stoichiometry. This behavior may be associated with the distinct coordination environments and reactivities of Zn^2+^ and In^3+^ during QD formation, which can affect their incorporation into the growing nanocrystals.^22^ The C 1s, O 1s, and N 1s spectra provided in the Supporting Information further confirm the presence of the surface ligands. The higher relative contribution of S, O, N, and C reflects the presence of surface ligands and the surface-sensitive nature of XPS, which enhances the signal from ligand-derived species compared to the inorganic core.

### 3.4 Structural Characterization of the TQDs

To provide structural evidence supporting the optical, electronic, and surface characterization results, complementary structural analyses were performed. XRD patterns of AIS, CIS, and ZIS QDs are displayed in Figure 5a–c. The diffraction pattern peaks of AIS QDs (27.1°, 46.1°, and 54.1°) and CIS QDs (27.7°, 47.2°, and 54.6°) are related to the (112), (204), and (312) planes, which can be indexed to the tetragonal chalcopyrite structure (Figure 5d,e). These reflections are consistent with reference patterns reported for AIS (JCPDS No. 00–025–1330) and CIS (JCPDS No. 01–085–1575), respectively.^5,27^

**Figure 5:**
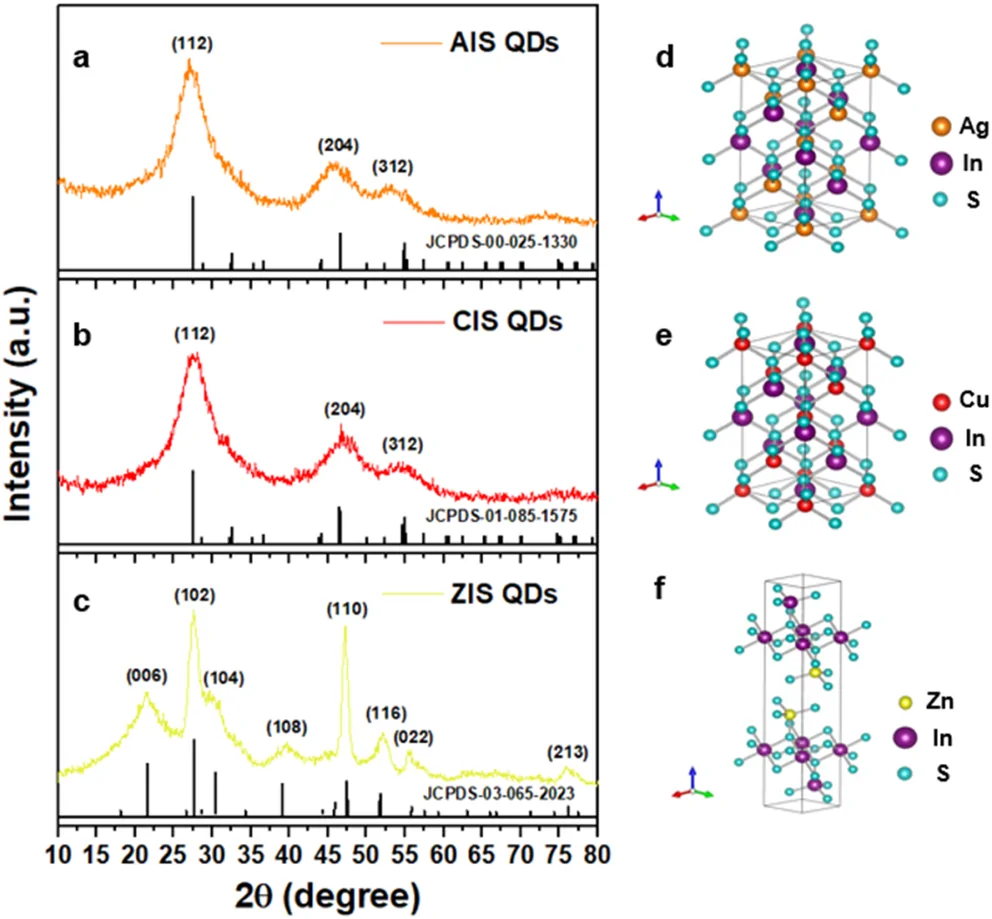
XRD patterns of (a) AIS, (b) CIS, and (c) ZIS QDs. Crystal structures of (d) tetragonal chalcopyrite AIS, (e) tetragonal chalcopyrite CIS, and (f) hexagonal ZnIn_2_S_4_ (wurtzite-like). The structures were produced using the VESTA software.

ZIS can crystallize in three different phases: cubic, hexagonal, and rhombic, among which the hexagonal phase exhibits photoluminescence and photoconductivity, making it an active photocatalytic material.^65^ The diffraction pattern of the ZIS QDs (21.57°, 27.66°, 29.94°, 39.58°, 47.30°, 52.12°, 55.42°, and 75.96°) corresponds to the hexagonal phase (JCPDS card No. 01–078–1274) (Figure 5c,5f). It exhibits a layered architecture characterized by a repetitive atomic stacking sequence of S–Zn–S–In–S–In–S. Zn atoms and half of the indium atoms adopt tetrahedral coordination with surrounding sulfur atoms within this structure, whereas the remaining indium atoms are situated in octahedral coordination sites with sulfur.^66^

The morphologies and crystallinities of AIS, CIS, and ZIS QDs were then investigated using high-resolution transmission electron microscopy (HRTEM) (Figure 6). TEM images confirm the successful formation of well-dispersed, nearly spherical nanoparticles for all three systems. The particle size distributions (Figure 6c,6f,6i) reveal average diameters of 5.57 ± 1.51 nm for AIS, 5.99 ± 1.08 nm for CIS, and 4.14 ± 0.83 nm for ZIS QDs, indicating relatively narrow size distributions. While AIS and CIS QDs exhibit similar sizes, ZIS QDs are slightly smaller and more uniform, suggesting differences in nucleation and growth behavior under the respective synthesis conditions.

**Figure 6:**
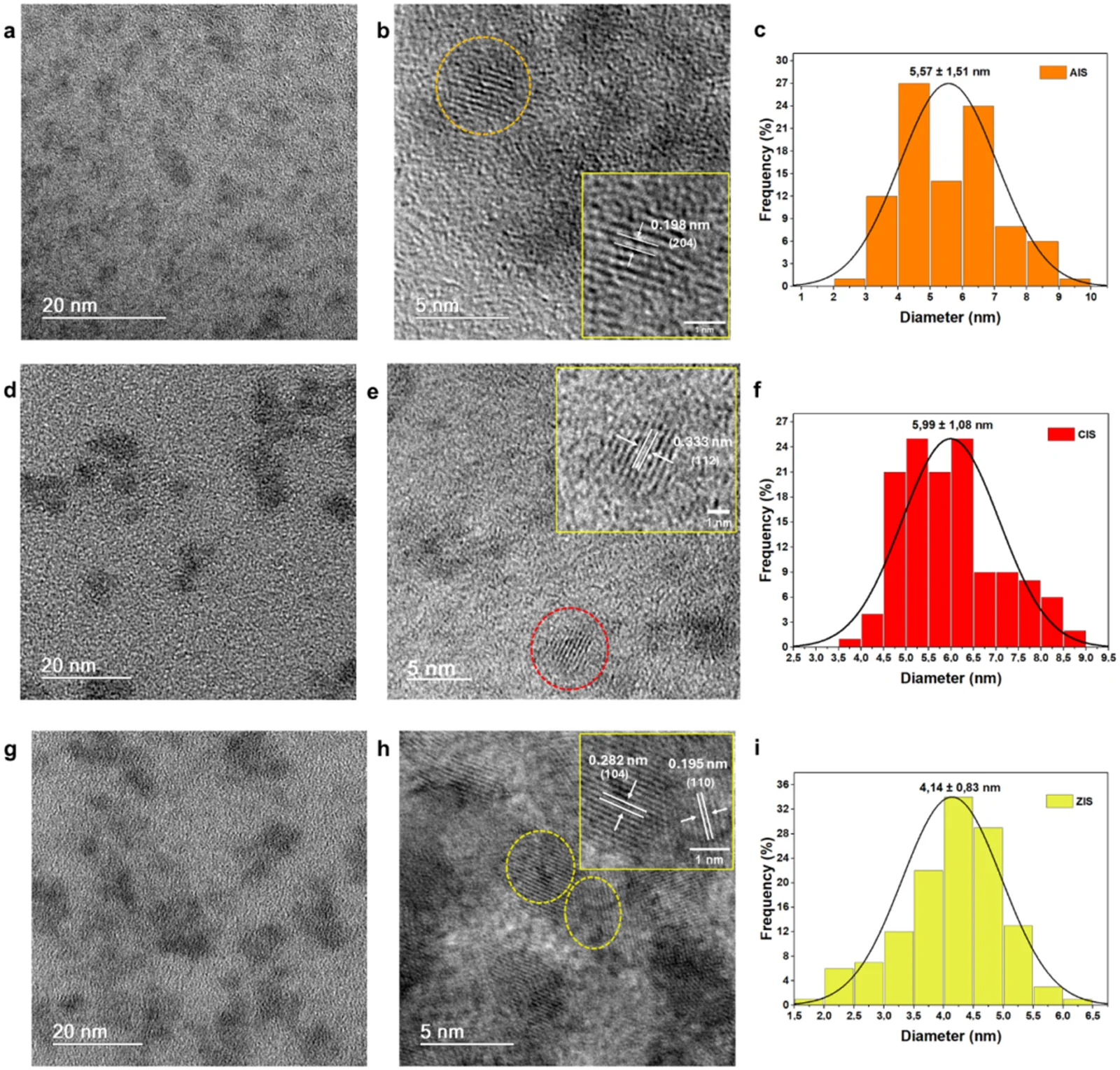
TEM and HRTEM images showing interplanar distance of AIS QDs (a, b), CIS QDs (d, e), and ZIS QDs (g, h), as well as their respective size distribution (c, f, i).

HRTEM images (Figure 6b,e,6h) show clear lattice fringes for all samples with interplanar spacings of 0.198 nm for AIS and 0.333 nm for CIS corresponding to the (204) and (112) planes of the tetragonal chalcopyrite structure, respectively. In the case of ZIS QDs, lattice spacings of 0.282 and 0.195 nm are consistent with the (104) and (110) planes of the hexagonal ZnIn_2_S_4_ phase. The corresponding SAED patterns, provided in the Supporting Information, exhibit well-defined diffraction rings indexed to the characteristic (112), (204), and (312) planes for AIS and CIS QDs, and the (006), (104), and (110) planes for ZIS QDs.

Table S4 compares the average particle sizes, crystal structures, and synthesis methods reported in the literature for AIS, CIS, and ZIS QDs with those obtained in this work. The comparison highlights the wide variability in particle sizes resulting from differences in synthesis conditions, precursor reactivity, and growth kinetics among distinct synthetic approaches. In contrast, the observed crystal structures are consistent with those commonly reported for these ternary QD systems.

Overall, the combined results obtained from XRD, HRTEM, SAED, and XPS consistently support the successful formation of the intended chalcopyrite (AIS and CIS) and hexagonal (ZIS) TQDs, without evidence of secondary crystalline phases.

FTIR spectra, provided in the Supporting Information, were used to investigate the surface composition and ligand binding of the QDs. For AIS and CIS QDs, the spectra retain the main features of MPA; however, the disappearance of the S–H stretching band (∼2564 cm^–1^) indicates coordination of the thiol group to the metal cations. Furthermore, the shift of the carboxylate stretching bands to 1564 and 1394 cm^–1^, corresponding to asymmetric and symmetric COO^–^ vibrations, confirms deprotonation of the carboxylic acid groups and their conversion to carboxylate species. Considering the strong affinity of thiol groups toward coinage-metal chalcogenide surfaces, the interaction of MPA with the QDs is commonly proposed to occur through the sulfur atom, leaving the carboxylate group exposed to the aqueous medium.^67^ For ZIS QDs, a similar absence of the S–H stretching bands (∼2676 and 2566 cm^–1^) confirms the interaction of TGA with the QD surface. In addition, the C=O and C–O stretching bands of TGA (1706 and 1286 cm^–1^) are shifted to lower wavenumbers (1640 and 1230 cm^–1^), indicating changes in the chemical environment upon binding. The presence of characteristic bands associated with gelatin, such as N–H (∼1547 cm^–1^) and O–H (∼3325 cm^–1^), further confirms the interaction between TGA, gelatin, and the ZIS QDs. Overall, these results demonstrate the successful functionalization of the QDs surfaces with the respective ligands.^21^ Based on the FTIR analysis, a schematic representation of the ligand binding configuration on the QDs surface is proposed in Figure 7. In the case of ZIS QDs, the schematic also includes the presence of gelatin (represented in pink), illustrating its interaction with the TGA-capped QDs surface.

**Figure 7:**
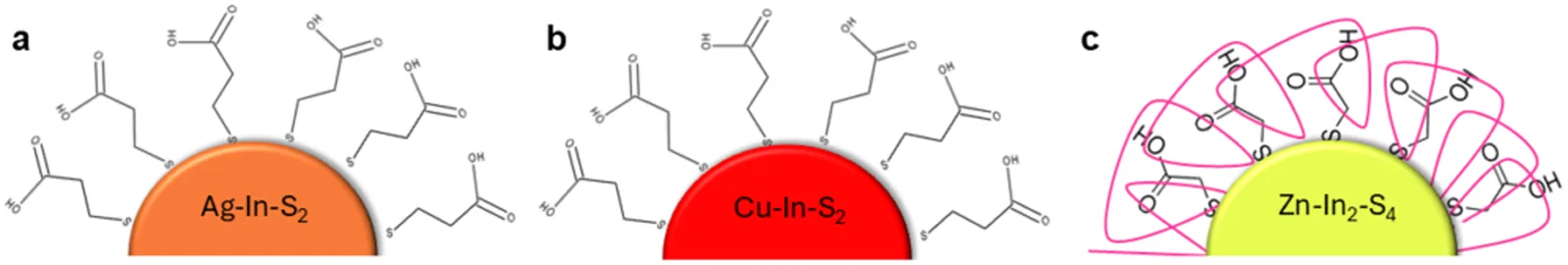
Scheme of the proposed ligand binding on the surface of (a) CIS QDs with MPA, (b) AIS QDs with MPA, and (c) ZIS QDs with TGA and gelatin (pink).

### 3.5 *In Vitro* Analysis of the Cytotoxicity of the TQDs

Following the comprehensive physicochemical characterization, the cytotoxicity of the synthesized QDs was evaluated through in vitro cytotoxicity assays. Although Cd-free quantum dots are generally considered safer alternatives to Cd-based nanomaterials, several studies have shown that they can still cause adverse biological effects, especially when used in their core-only form.^68,69^ For example, Kays et al.^70^ reported that CIS QDs showed *in vitro* cytotoxicity comparable to that of CdSe QDs, highlighting the need for further investigation into their biocompatibility.

The MTT assay is a widely used colorimetric method to assess cell viability. It is based on the ability of metabolically active cells to reduce the yellow tetrazolium salt MTT [3-(4,5-dimethylthiazol-2-yl)-2,5-diphenyltetrazolium bromide] into insoluble purple formazan crystals through mitochondrial enzymatic activity. The amount of formazan formed is directly proportional to the number of viable cells and can be quantified by spectrophotometry after solubilization. The cytotoxicity assays were performed using freshly prepared QD dispersions immediately after sample preparation. Throughout the experimental period, the dispersions remained visually homogeneous, with no visible precipitation or aggregation. The assay was performed using QDs at concentrations ranging from 20 to 150 μg mL^–1^. No significant reduction in cell viability was observed, even at the highest concentrations tested (Figure 8a–c). Morphological analysis of Chinese hamster ovary (CHO) cells before and after treatment is provided in the Supporting Information. These observations corroborated the cytotoxicity results, indicating no apparent cytotoxic effects from the QDs under our experimental conditions. Statistical analysis among groups (Control, 20, 80, and 150 μg mL^–1^), performed using one-way ANOVA followed by Tukey’s multiple-comparison test, revealed no statistically significant differences among the tested groups (*p* > 0.05), indicating that the observed variations among the means can be attributed to random variation rather than to treatment effects. Therefore, the treatment did not induce detectable cytotoxic effects under the experimental conditions evaluated. Specifically, no detectable cytotoxic effects were observed in CHO cells exposed to AIS, CIS, and ZIS QDs for 24 h at concentrations ranging from 20 to 150 μg mL^–1^. However, it should be noted that the MTT assay primarily provides an indication of cellular metabolic activity and, by itself, does not establish overall biocompatibility or exclude other cellular responses. Therefore, these findings should not be extrapolated to other cell types, exposure periods, or concentrations without further investigation.

**Figure 8:**
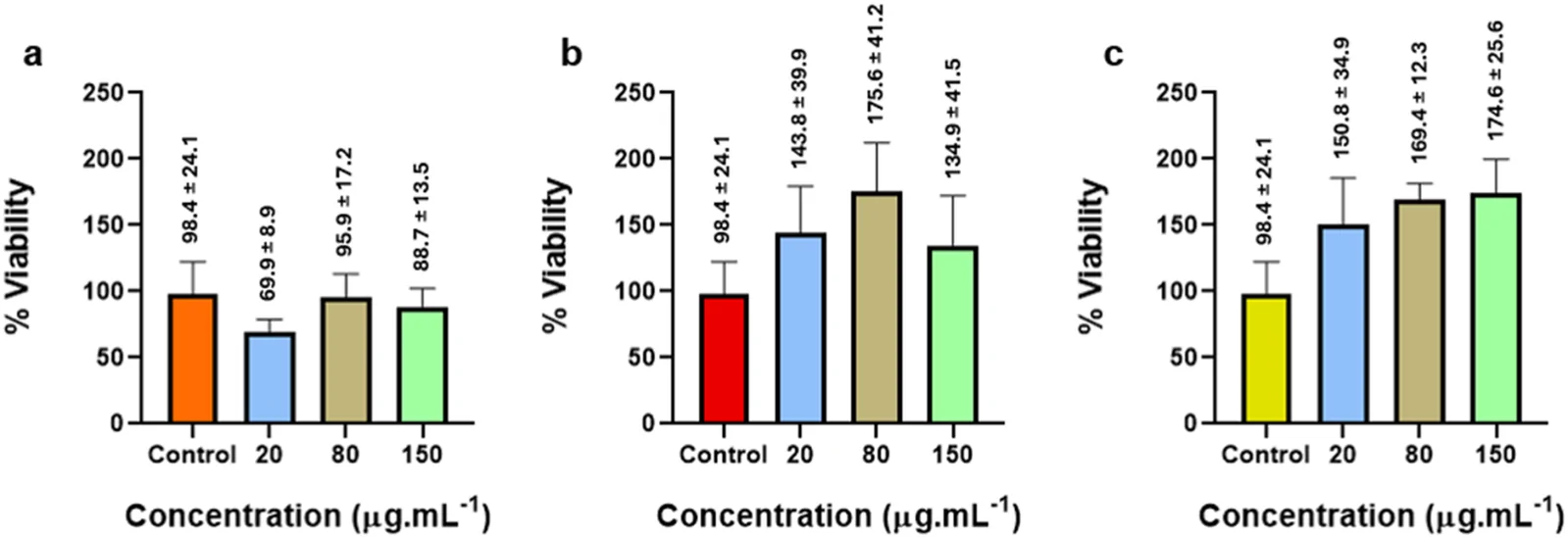
Cell viability in different concentrations of (a) AIS, (b) CIS, and (c) ZIS QDs.

The absence of detectable cytotoxicity observed for AIS, CIS, and ZIS QDs under the conditions evaluated contrasts with the cytotoxic effects reported for some Cd-free QD systems, including core-only CIS QDs.^68–70^ This comparison suggests that the biological response of QDs is not determined solely by their elemental composition, but may also depend on factors such as surface chemistry, colloidal stability, particle size, defect density, and exposure conditions. Therefore, the present results indicate low cytotoxicity of the synthesized AIS, CIS, and ZIS QDs under the specific experimental conditions investigated.

## 4 Conclusions

In this study, the AIS, CIS, and ZIS TQDs were synthesized by environmentally friendly one-pot aqueous routes and systematically compared under a unified comparative framework. The adopted approach enabled a direct evaluation of the influence of cation composition on the structural, optical, electronic, and biological properties of environmentally friendly TQDs.

Although the three systems exhibited similar crystal structures and nanometric dimensions, their optical and electronic properties differed markedly as a function of cation composition. The three materials exhibited broad absorption in the visible region and size/composition-dependent photoluminescence, with bandgaps tuned between 1.96 and 2.42 eV. The second-derivative method proved more reliable than the Tauc approach for determining bandgap in defect-rich systems and for characterizing Urbach tails. Electrochemical studies, including cyclic voltammetry and Mott–Schottky analyses, validated the band edge positions and revealed systematic differences between optical and electrochemical bandgaps, attributed to excitonic effects and surface influences. Furthermore, the good agreement between Mulliken electronegativity calculations and experimental data supports the use of this approach as a qualitative descriptor of band alignment. Electrochemical impedance and Mott–Schottky analyses indicate differences in charge transfer resistance and band alignment, confirming that cation identity (Ag, Cu, Zn) plays a key role in governing interfacial charge transport and recombination pathways.

The XPS analyses provided further insights into the composition and surface states. AIS and CIS QDs exhibited pronounced Ag and Cu deficiencies, respectively. In contrast, ZIS QDs displayed a Zn:In ratio close to the nominal stoichiometric composition expected for ZnIn_2_S_4_, while the sulfur signal was dominated by contributions from the organic ligands (TGA and gelatin), underscoring the significant role of surface chemistry and stabilizers in the synthesis of this challenging material.

Finally, cytotoxicity assays showed no detectable cytotoxic effects of AIS, CIS, and ZIS QDs toward cells CHO under the specific experimental conditions evaluated, including 24 h of exposure and concentrations ranging from 20 to 150 μg mL^–1^. These findings provide an initial indication of cytocompatibility under the tested conditions, but further studies using additional cellular models, exposure periods, concentrations, and complementary biological assays are required to assess the broader biological compatibility of these QDs.

Overall, this work demonstrates that the electronic structure and functional properties of TQDs are strongly governed by cation composition, defect states, and surface chemistry. By combining optical and electrochemical approaches within a unified framework, this study provides insight into how band alignment and recombination pathways can be tuned in aqueously synthesized TQDs. These findings establish a direct correlation between composition and functionality, offering practical guidance for the rational design of cadmium-free quantum dots for applications in optoelectronics, photocatalysis, and biorelated technologies.

## Supplementary Material



## Data Availability

Data will be made available on request.

## Notes

Structural, optical, and electrochemical characterization of AIS, CIS, and ZIS QDs. These data comprise synthesis schematics, temperature-dependent UV–vis and photoluminescence spectra, detailed bandgap determination (Tauc plots and second derivative analysis), and the equations used for τ fitting and PLQY calculations; further information includes Mulliken electronegativity calculations, conversion of electrochemical potentials from Ag/AgCl to NHE and absolute vacuum scale, and additional electrochemical parameters derived from Mott–Schottky analysis; complementary XPS data, including high-resolution spectra and survey spectra, are also provided, along with compositional analysis; additional structural characterization (SAED patterns, particle size comparison with literature data, and FTIR spectra) is presented; finally, supporting biological data includes morphological analysis of CHO cells before and after exposure to QDs, confirming the observations (PDF)
