# Supplementary material for: Comparative Study of Properties and Cytotoxicity of Eco-Friendly AgInS2, CuInS2, and ZnIn2S4 Quantum Dots under a Unified Experimental Framework
Source: ACS Omega. 2026 Oct 21;11(43):67853–67. doi: 10.1021/acsomega.6c09767 (PMC13737961; doi:10.1021/acsomega.6c09767)
Supplement: Supplementary file 1 [file ao6c09767_si_001.pdf]

Supporting Information

Comparative Study of Properties and Cytotoxicity of Eco-Friendly $AgInS_2$, $CuInS_2$, and $ZnIn_2S_4$ Quantum Dots under a Unified Experimental Framework

*Cecília B. da Silva[1], Letícia R. C. Cunha[1], Paulo H. A. Campos-Junior[2], Arnaldo C. Pereira[3], Dmitry Aldakov[4], Marco A Schiavon[1]**

[1]Materials Chemistry Research Group, Department of Natural Sciences, Federal University of São João del-Rei, Dom Bosco Campus, São João del-Rei, MG 36301-160, Brazil.

[2]Laboratory for Biology of Fertility Research, Department of Natural Sciences, Federal University of São João del-Rei, Dom Bosco Campus, São João del-Rei, MG 36301-160, Brazil.

[3]Polymer and Electroanalytics Research Group, Department of Natural Sciences, Federal University of São João del-Rei, Dom Bosco Campus, São João del-Rei, MG 36301-160, Brazil.

[4]Université Grenoble Alpes, CNRS, CEA, IRIG, SYMMES, STEP, 38000, Grenoble, France.

*Email: schiavon@ufsj.edu.br

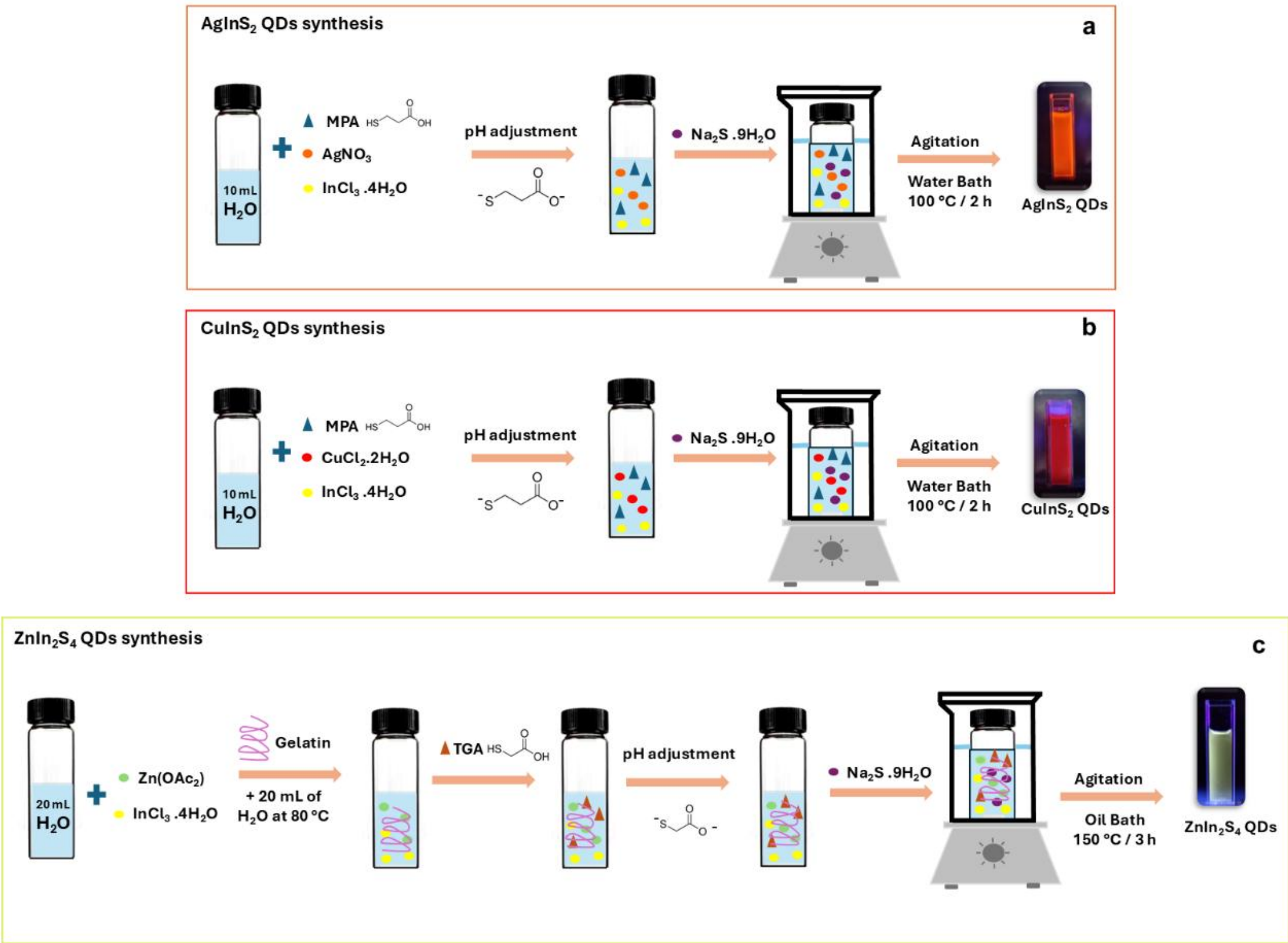

Figure S1. Schematic model for synthesizing a) AIS, b) CIS, and c) ZIS QDs.

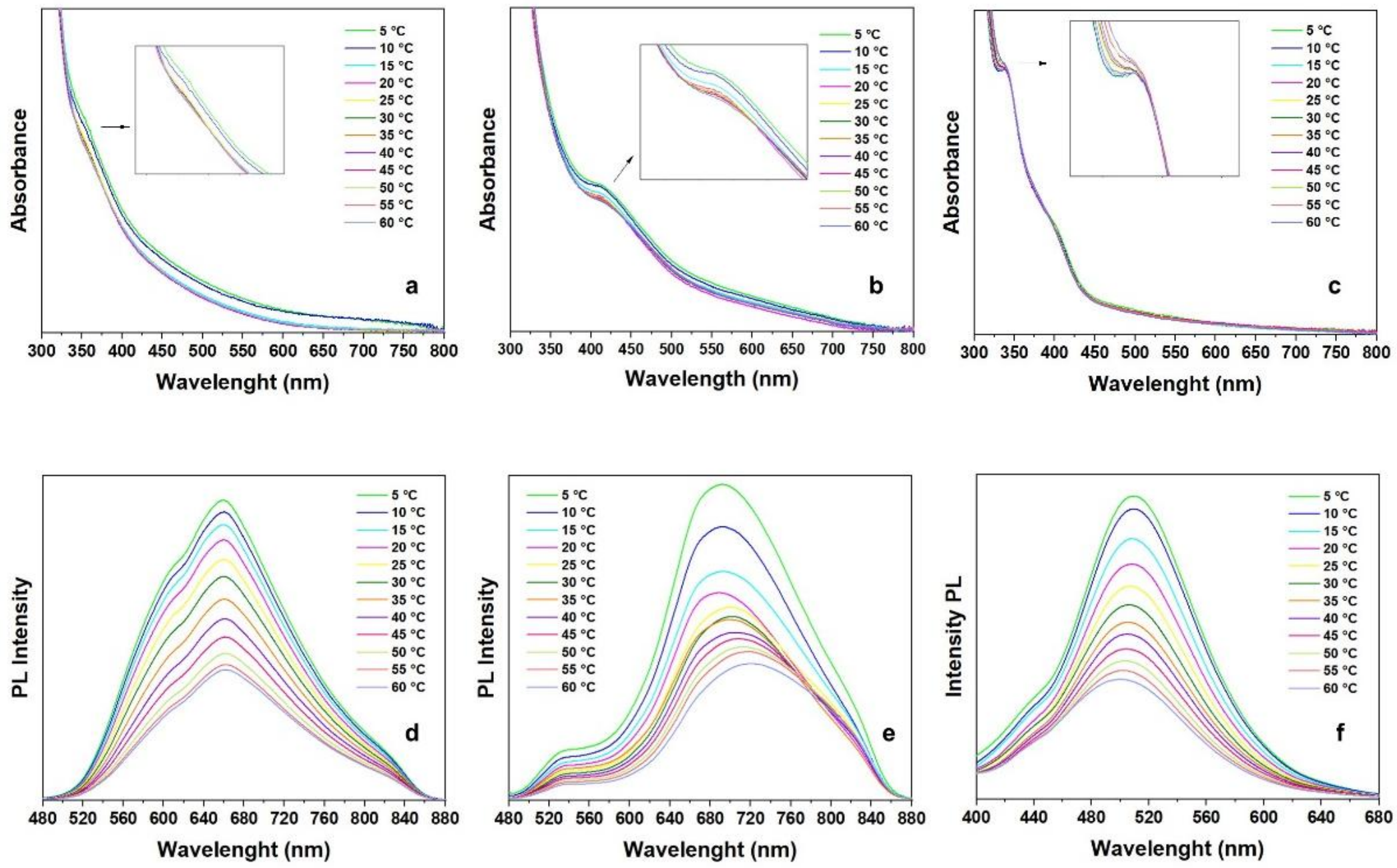

Figure S2. UV-Vis absorption spectra of (a) AIS, (b) CIS, and (c) ZIS QDs; and PL spectra of (d) AIS, (e) CIS, and (f) ZIS QDs at different temperatures.

Biexponential function to fit the time-resolved photoluminescence (τ):

$$F(t) = A_1 \exp(-t/\tau_1) + A_2 \exp(-t/\tau_2) \qquad \text{Eq. S1}$$

where τ_1 and τ_2 are the decay times, and A_1 and A_2 are the fractional contributions of the two components, respectively.

Average lifetime equation:

$$\tau_{average} = (A_1\tau_1^2 + A_2\tau_2^2) \,/\, (A_1\tau_1 + A_2\tau_2) \qquad \text{Eq. S2}$$

The radiative (k_r) and non-radiative (k_{nr}) decay rate constants were calculated from the PLQY and average PL lifetime (τ) according to $k_r = \mathrm{PLQY}/\tau$ and $k_{nr} = (1 - \mathrm{PLQY})/\tau$, respectively.

PLQY equation:

$$PLQY\ (\%) = PLQY_{(standard)} = \left({Slope_{QDs}}/{Slope_{standard}}\right)\left({\eta_{QDs}}/{\eta_{standard}}\right) \qquad \text{Eq. S3}$$

The term slope refers to the relationship between the absorbance (at the excitation wavelength) and the integrated area of photoluminescence (of the QDs and the standard). η is the refractive index of the medium.

Tauc plot equation:

$$\alpha h\nu = B(h\nu - E_g)^{n} \qquad \text{Eq. S4}$$

Here, B is a constant, h is Planck's constant, E_g refers to the optical bandgap energy, and n is the factor linked to the electron transitions regime[1].

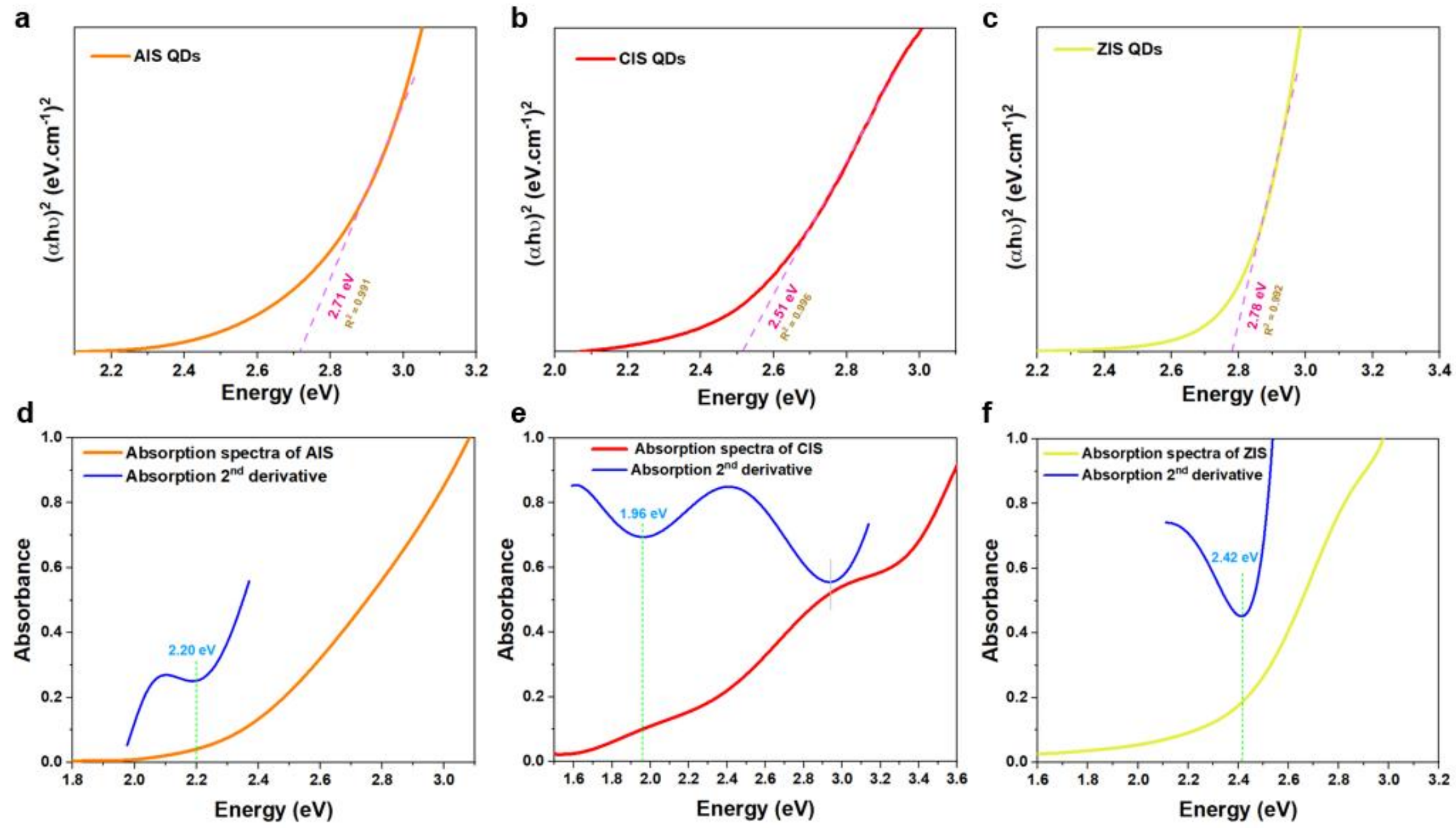

Figure S3. (a-c) Tauc plots used to estimate the bandgap energies of (a) AIS, (b) CIS, and (c) ZIS QDs. (d-f) Corresponding UV-Vis absorption spectra and their second derivatives (d^2A/dE) for (d) AIS, (e) CIS, and (f) ZIS QDs, highlighting the absorption edge used for bandgap determination.

Table S1 – Comparison of bandgap calculation methods and synthesis approaches for AIS, CIS, and ZIS QDs reported in the literature and obtained in this work

Samples	Calculation method	Bandgap (eV)	Synthesis method	References
AIS	Tauc Plot	2.70	Microwave radiation	2
	Second Derivative	2.58	Hot-injection method.	3
	Tauc Plot	2.05	Hot-injection method	4
	Tauc Plot	1.73	One-pot in aqueous media	5
	Tauc Plot	1.55	One-pot in aqueous media	6
	Second Derivative	2.20	One-pot in aqueous media	This work
CIS	Tauc Plot	1.81	Hydrothermal Route	7
	Tauc Plot	1.92	One-pot in aqueous media	8
	Tauc Plot	1.93	Hydrothermal Route	9

	Tauc Plot	2.20	Hot-injection method	10
	Tauc Plot	1.6	Hot-injection method	11
	Second Derivative	1.96	One-pot in aqueous media	This work
**ZIS**	Tauc Plot	2.49	Hydrothermal Route	12
	Tauc Plot	2.56	Hydrothermal Route	13
	Tauc Plot	2.59	Hydrothermal Route	14
	Tauc Plot	3.12	Hydrothermal Route	15
	Second Derivative	2.42	One-pot in aqueous media	This work

Mulliken electronegativity calculations

The Mulliken absolute electronegativity (χ) of each QD was estimated from its nominal stoichiometric composition using tabulated values of electron affinity (EA) and first ionization potential (IP) of the constituent elements, expressed in electronvolts (eV). Initially, the absolute electronegativity of each constituent atom was calculated according to Equation S5.

$$\chi_{atom} = \frac{EA+IP}{2} \quad \text{Eq. S5}$$

The effective electronegativity of each semiconductor was then calculated as a stoichiometrically weighted average of the atomic electronegativities of all constituent elements, according to Equation S6:

$$\chi_{sample} = \frac{\sum_{i=1}^{n}(\chi_i \cdot N_i)}{\sum_{i=1}^{n} N_i} \quad \text{Eq. S6}$$

where χ_i is the absolute electronegativity of each constituent atom and N_i is the stoichiometric coefficient of element $i$in the chemical formula of the sample.

For AIS and CIS QDs, the calculation was performed considering the three constituent elements of the semiconductor, as exemplified for the AIS system in Equation S7. For CIS QDs, the same procedure was applied by replacing $\chi_{Ag}$with χ_{Cu}.

$$\chi_{AIS} = \frac{(\chi_{Ag} .1)+ (\chi_{In} .1)+ (\chi_S .2)}{1+1+2} \quad \text{Eq. S7}$$

Similarly, for ZIS QDs, the calculation was performed considering the four constituent elements of the semiconductor, as shown in Equation S8.

$$\chi_{ZIS} = \frac{(\chi_{Zn}.1) + (\chi_{In}.2) + (\chi_{S}.4)}{1+2+4} \quad \text{Eq. S8}$$

Conversion of Redox Potentials from Ag/AgCl to NHE and Absolute Vacuum Scale

In photoelectrochemistry, the positions of the energy levels are typically related to the absolute vacuum scale (AVS), while the redox potentials (E_{ox} and E_{red}) are given relative to the normal hydrogen electrode (NHE) ($E^0_{NHE}(H^+/H_2) = 0$ V). Thus, the onset redox potential, measured by CV (relative to the Ag/AgCl reference electrode), was converted to the NHE and AVS scales using Equations S9 and S10:

$$E_{(vs.\ NHE)} = \left(E_{(vs.\ Ag/AgCl)} + 0.197\right)eV \quad \text{Eq. S9}$$

The value of 0 V in NHE is equivalent to −4.5 eV (298.15 K) in the AVS scale. Thus, $E_{(vs.\ AVS)}$ values were calculated according to Equation S10:

$$E_{(vs.\ AVS)} = -\left(E_{(vs.\ NHE)} + 4.50\right)eV \quad \text{Eq. S10}$$

Table S2 – Intercept potential (V_0), flat-band potential (V_{fb}), and calculated conduction (E_{CB}) and valence band (E_{VB}) edge positions of AIS, CIS, and ZIS QDs

Sample	V_0 (V)	V_{fb} (V)	$E_{CB(Ag/AgCl)}$ (eV)	$E_{CB/NHE}$ (eV)	$E_{VB/NHE}$ (eV)
**AIS**	-0.63	-0.66	-0.76	-0.56	1.64
**CIS**	-0.87	-0.90	-1.00	-0.80	1.16
**ZIS**	-0.60	-0.63	-0.73	-0.53	1.89

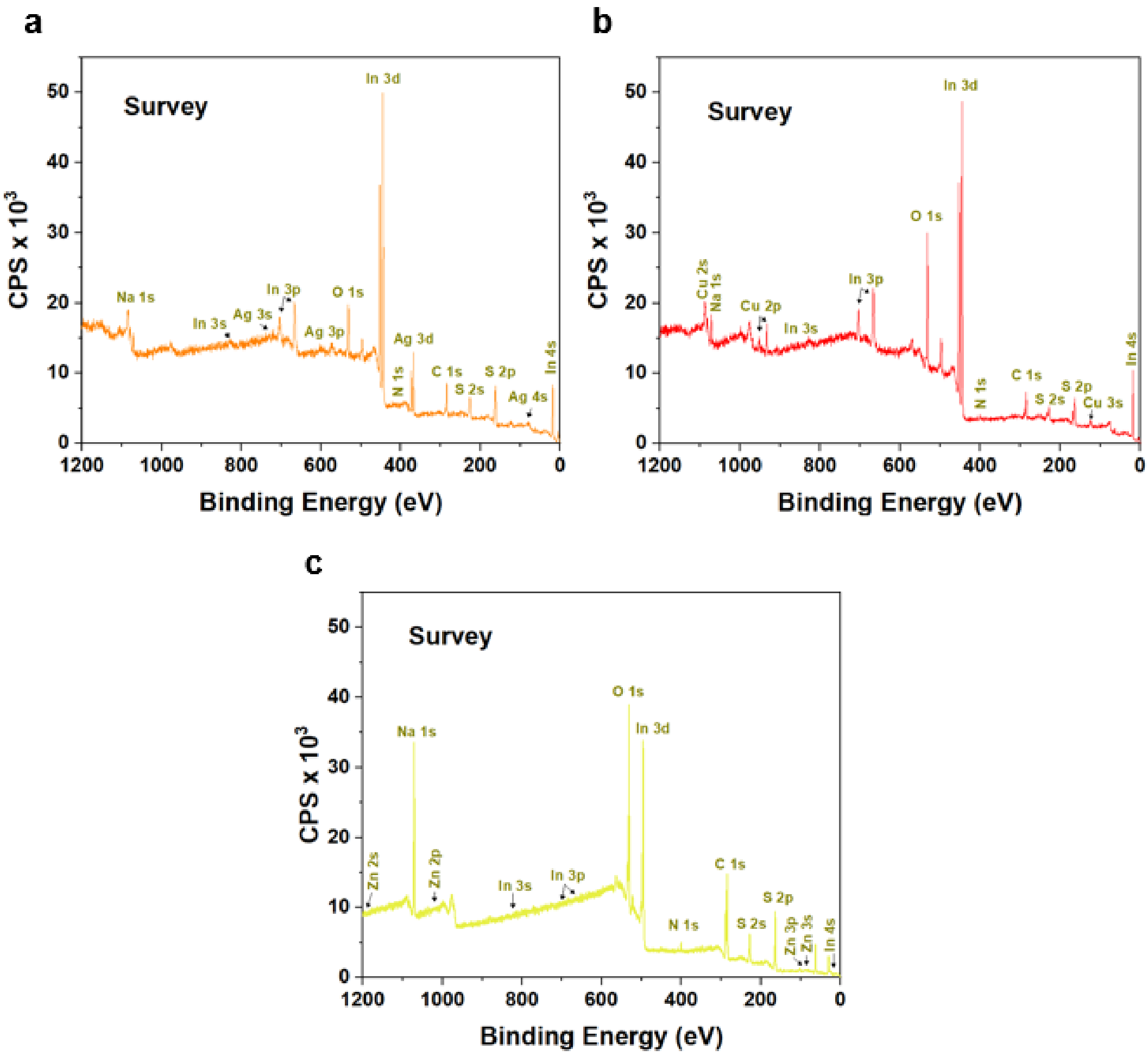

Figure S4. Survey spectra of (a) AIS QDs, (b) CIS QDs, and (c) ZIS QDs.

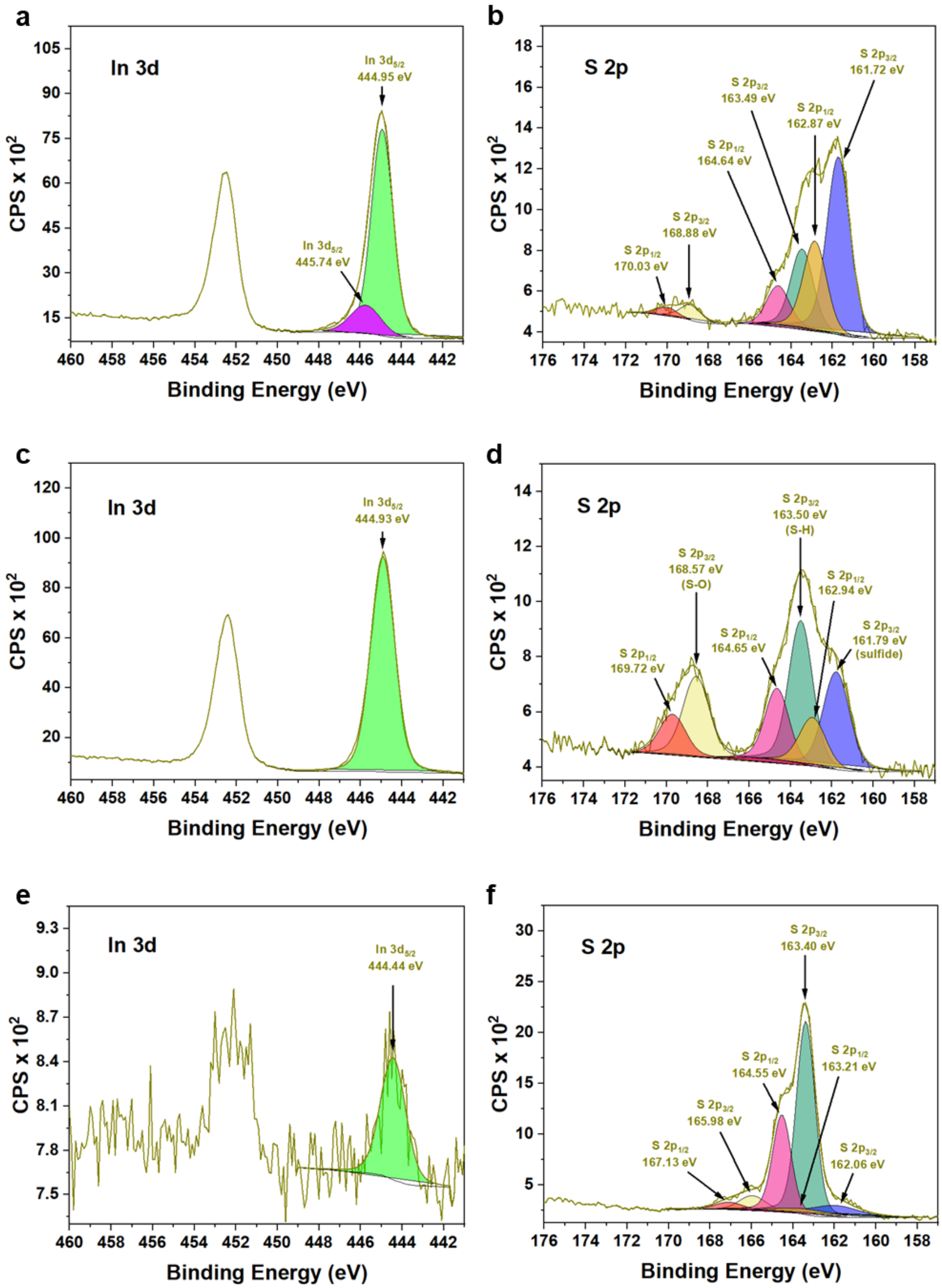

Figure S5. XPS spectra of the different elements in the AIS QDs (a) In 3d and (b) S 2p. XPS spectra of the different elements in the CIS QDs (c) In 3d and (d) S 2p. XPS spectra of the different elements in the ZIS QDs (e) In 3d and (f) S 2p.

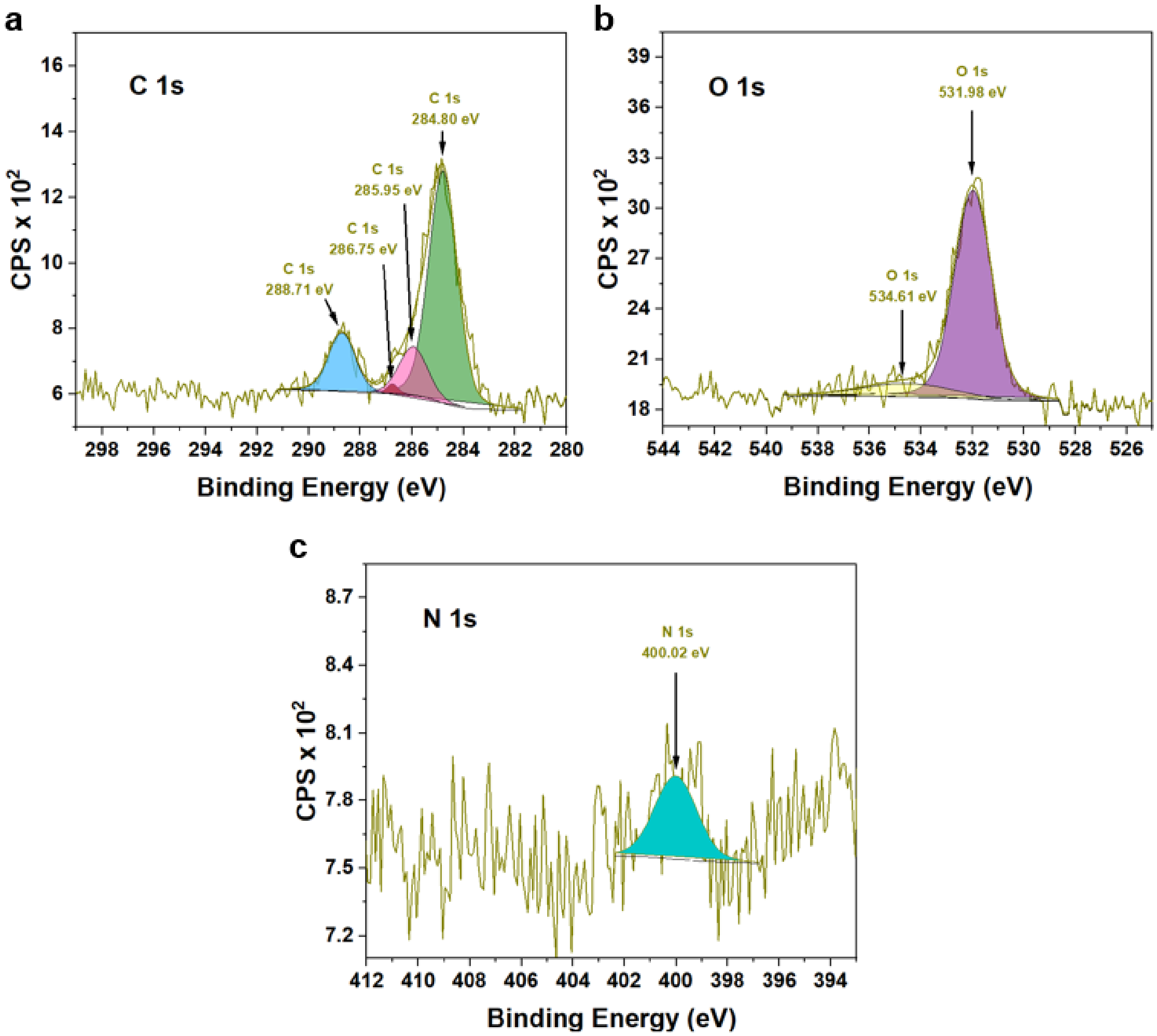

Figure S6. XPS spectra of the different elements in the AIS QDs. High-resolution X-ray photoelectron spectra of (a) C 1s, (b) O 1s, and (c) N 1s.

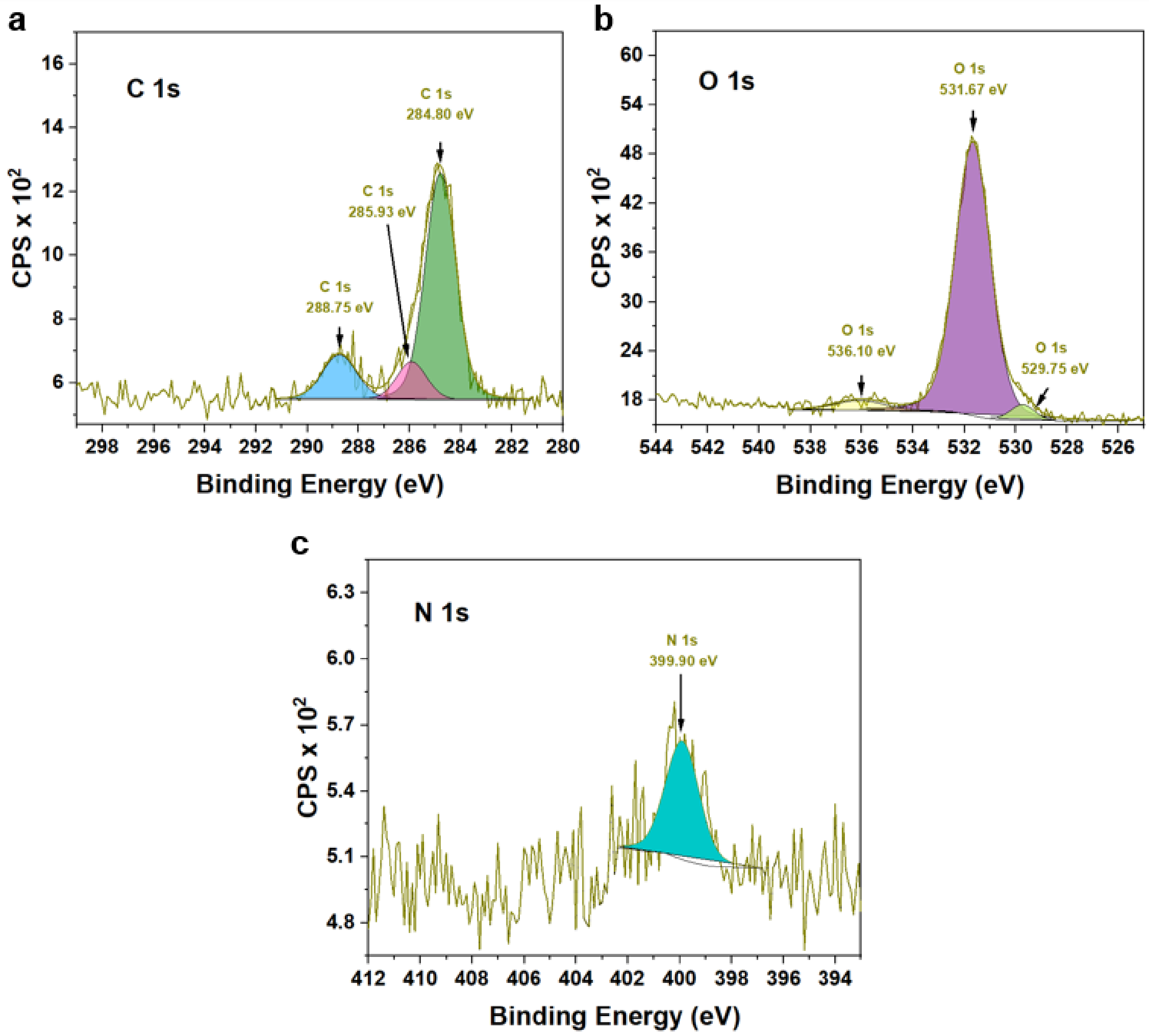

Figure S7. XPS spectra of the different elements in the CIS QDs. High-resolution X-ray photoelectron spectra of (a) C 1s, (b) O 1s, and (c) N 1s.

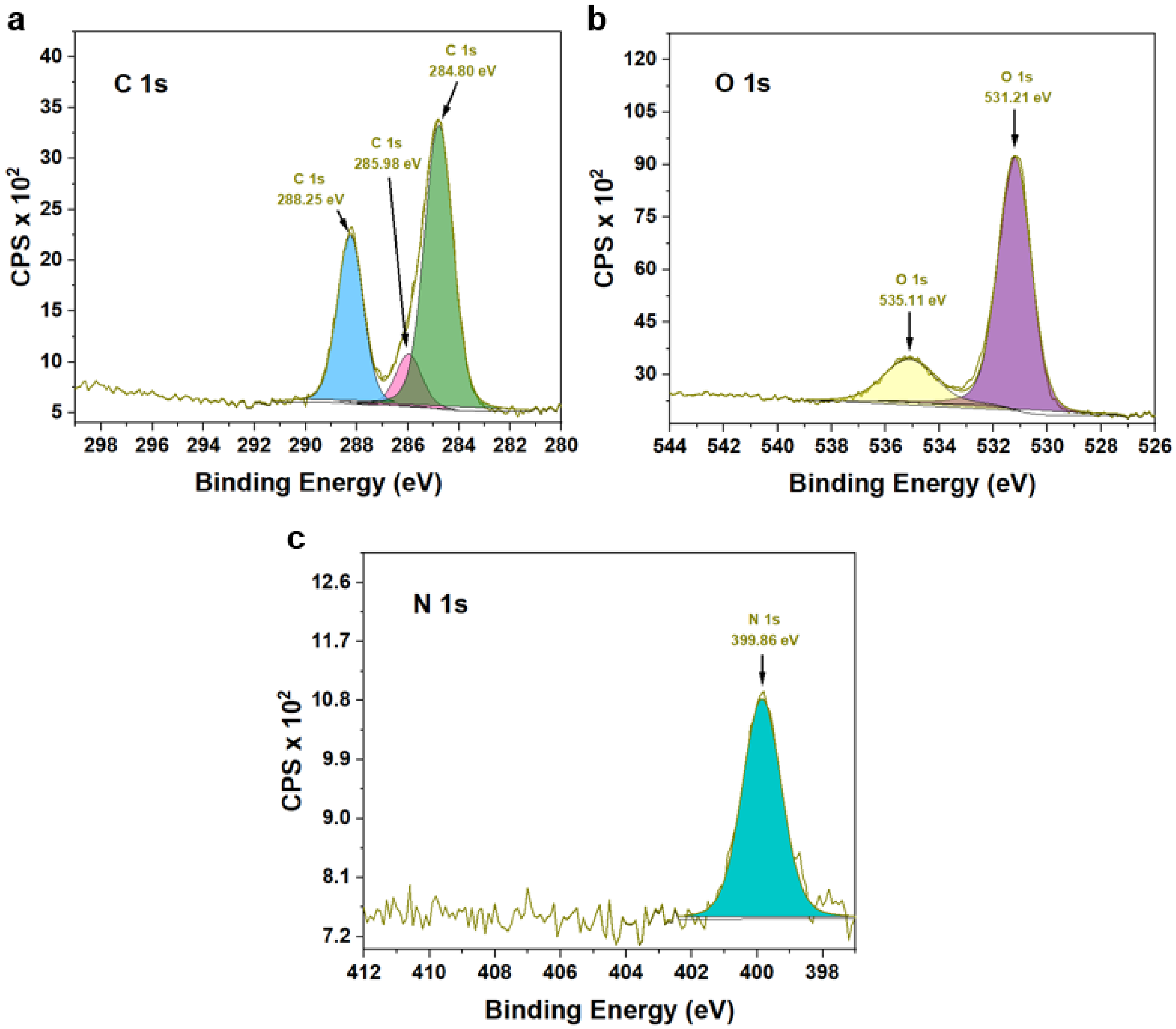

Figure S8. XPS spectra of the different elements in the ZIS QDs. High-resolution X-ray photoelectron spectra of (a) C 1s, (b) O 1s, and (c) N 1s.

Table S3 – Chemical composition normalized with respect to the metal cation, XPS analysis results

	**Ag**	**Cu**	**Zn**	**In**	**S**
**AIS**	1	-	-	4.5	9.7
**CIS**	-	1	-	13.4	24.1
**ZIS**	-	-	1	2.0	534.7

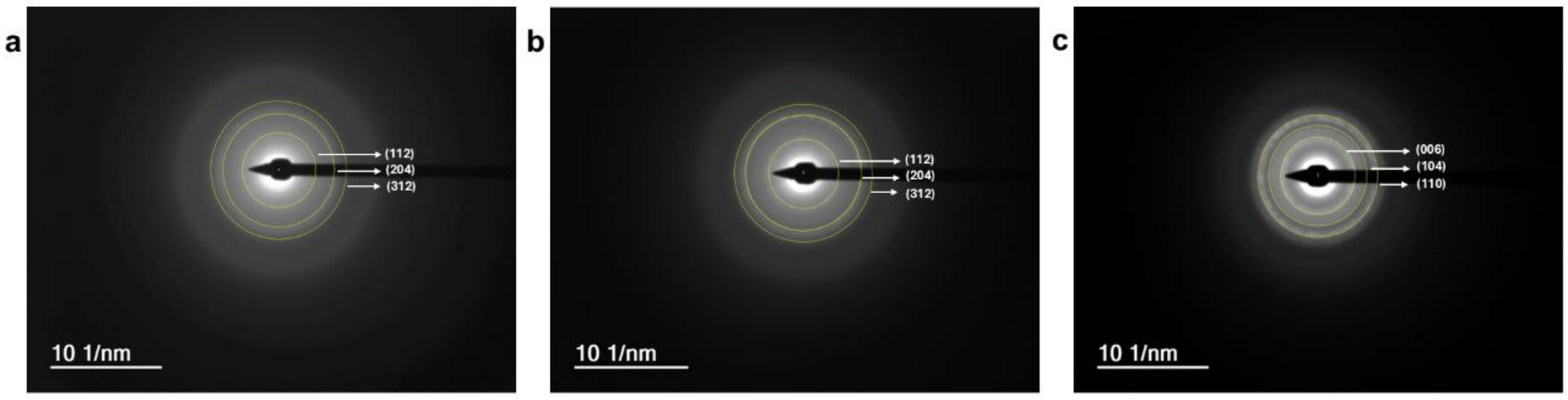

Figure S9. (a) SAED pattern of AIS QDs, (b) CIS QDs, and (c) ZIS QDs.

Table S4 – Comparison of crystal structure, particle size, and synthesis method for AIS, CIS, and ZIS QDs reported in the literature and obtained in this work

Samples	Crystal structure	Diameter (nm)	Synthesis method	References
**AIS**	Chalcopyrite	2.73	One-pot in aqueous media	16
		2.5	Microwave radiation	17
		~3.01–4.69	Hydrothermal method	18
		~3.35–4.68	Microwave-assisted hydrothermal method	19
		5.57 ± 1.51	One-pot in aqueous media	This work
**CIS**	Chalcopyrite	2 ± 0.5	One-pot in aqueous media	20
		~5–8	Hot injection in aqueous media	8
		4.5 ± 0.5	Hydrothermal Route	21
		3.2	One-pot in aqueous media	22
		5.99 ± 1.08	One-pot in aqueous media	This work
**ZIS**	Hexagonal	~3–7	Hydrothermal Route	15
		~5–15	Hydrothermal Route	23
		≈ 3	One-pot in aqueous media	24
		6.7 ± 1.3	One-pot in aqueous media	25
		4.14 ± 0.83	One-pot in aqueous media	This work

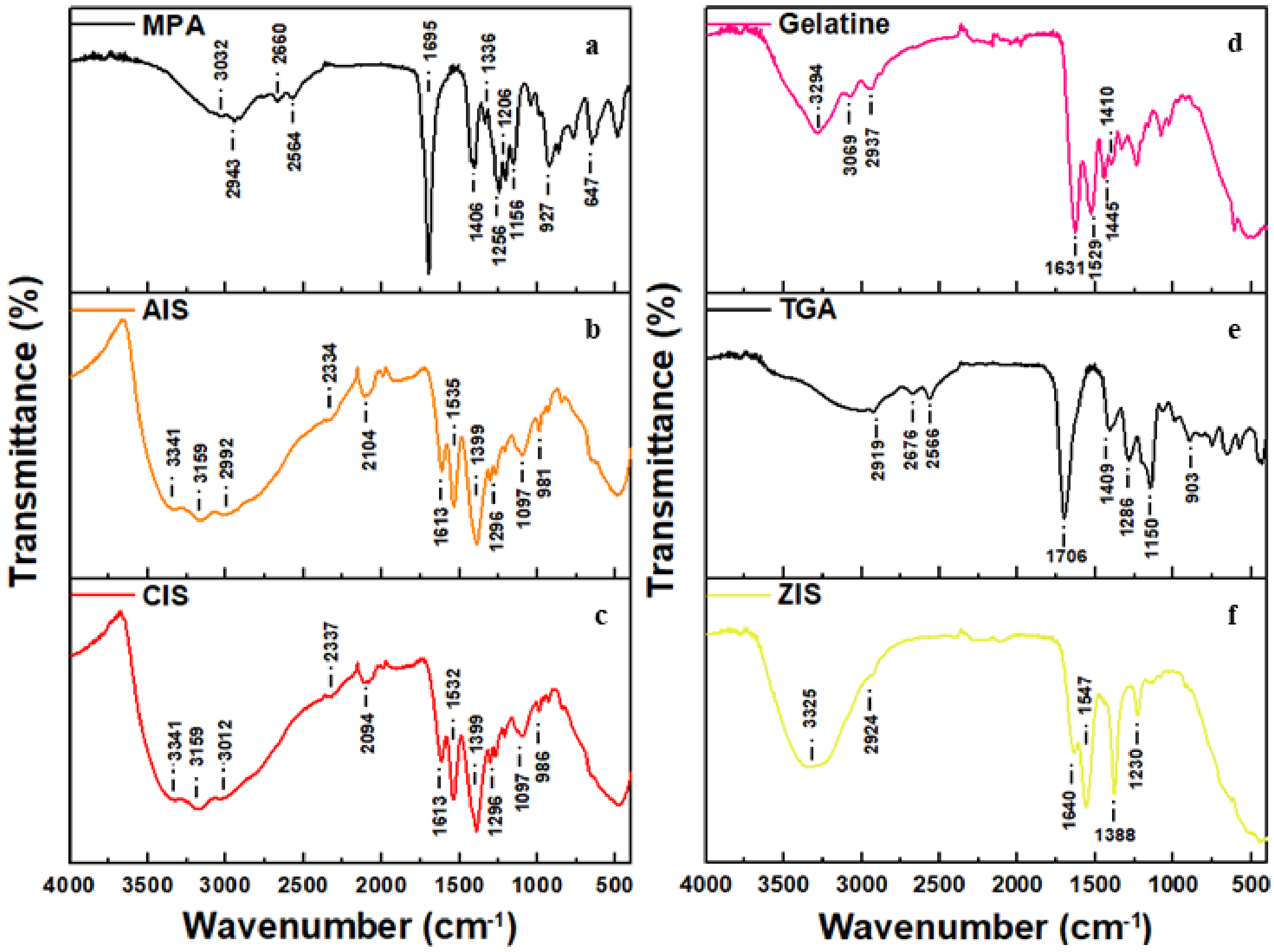

Figure S10. FTIR spectra of (a) pure MPA, (b) AIS QDs, (c) CIS QDs, (d) gelatin, (e) pure TGA, and (f) ZIS QDs.

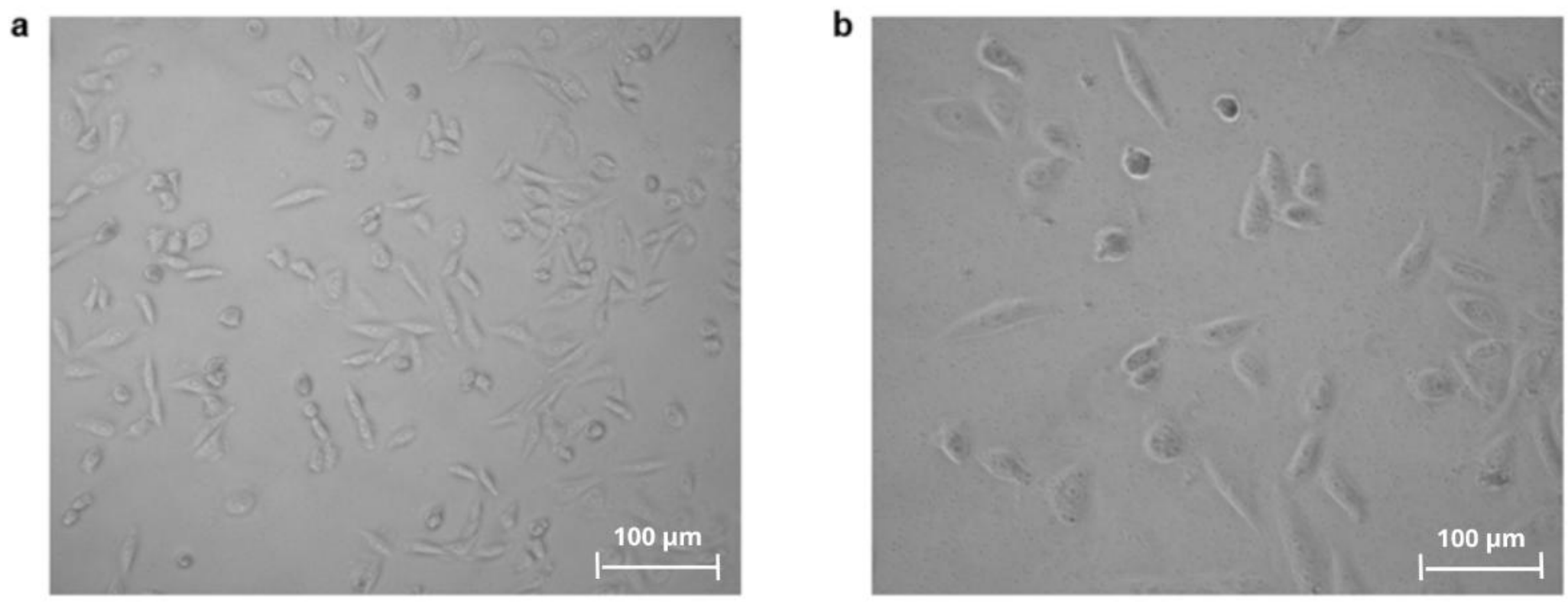

Figure S11. Morphological images of CHO cells before treatment with QDs at (a) 20x magnification and (b) 40x magnification.

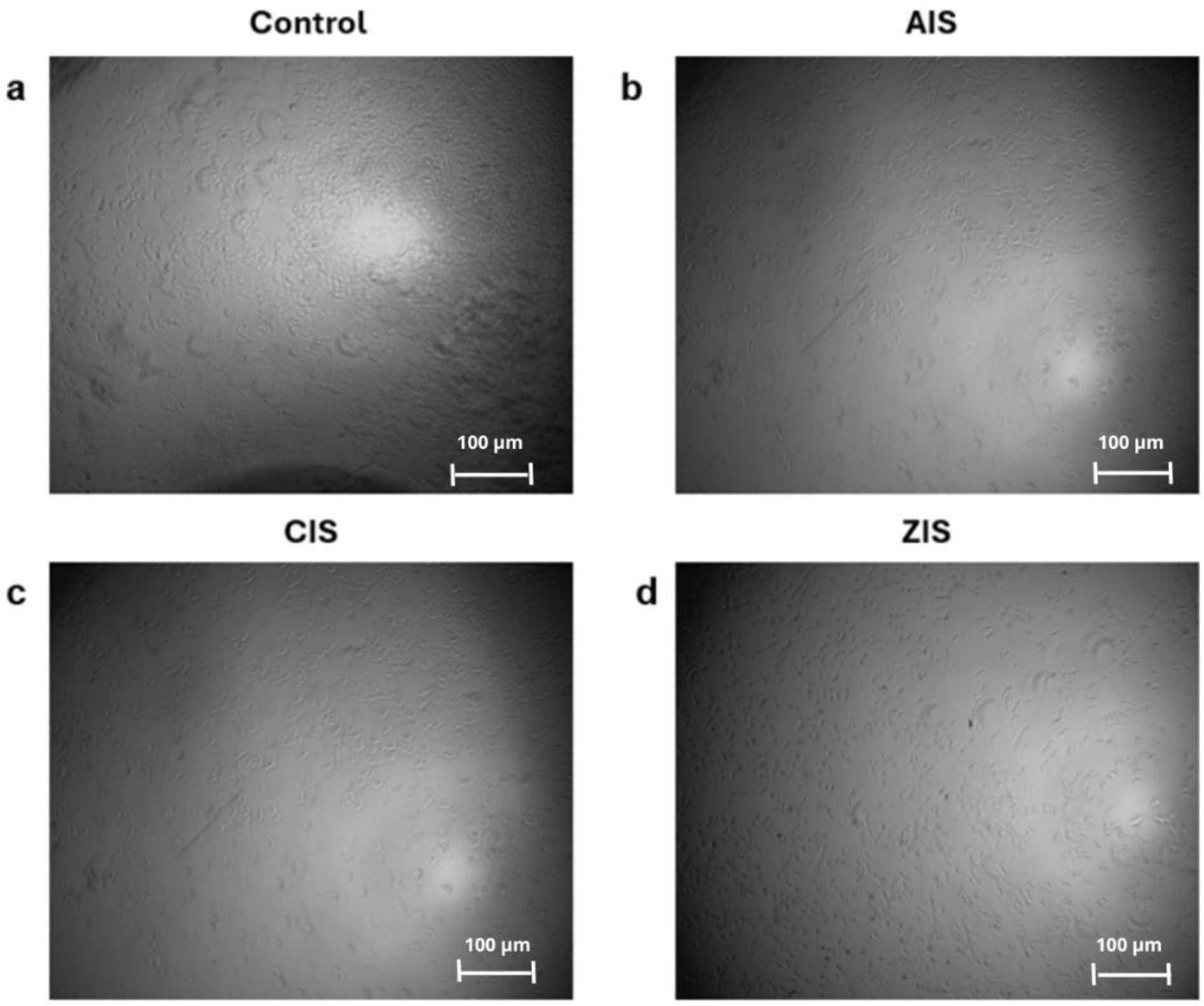

Figure S12. Morphological images of CHO cells treated with the concentration of 150 µg mL^{-1} QDs: (a) control (b), AIS QDs (c), CIS QDs, and (d) ZIS QDs. The images were taken at a scale bar (100 µm) and 20x magnification.

References

(1) Ebaid, A.; El-Hamalawy, A.; ElKholy, M.; Ebrahim, S.; El-Nady, J. Fabrication of Low-Cost Quantum Dot Sensitized Solar Cells (QDSSCs). *Advances in Basic and Applied Sciences* **2024**, *2* (1), 59–66. https://doi.org/10.21608/abas.2023.243952.1032.

(2) Mir, I. A.; Radhakrishanan, V. S.; Rawat, K.; Prasad, T.; Bohidar, H. B. Bandgap Tunable AgInS Based Quantum Dots for High Contrast Cell Imaging with Enhanced Photodynamic and Antifungal Applications. *Sci. Rep.* **2018**, *8* (1), 9322. https://doi.org/10.1038/s41598-018-27246-y.

(3) Kipkorir, A.; Chen, B. A.; Kamat, P. V. Anion-Driven Bandgap Tuning of AgIn(SxSe1-x)2 Quantum Dots. *ACS Nano* **2024**, *18* (41), 28170–28177. https://doi.org/10.1021/acsnano.4c07774.

(4) Shen, F.; Fan, Y. M.; Li, H.; Li, S. P.; Xu, M.; Dai, W. B. Photophysics and Photovoltaic Properties of Zn-Alloyed Ag-In-S Quantum Dots Sensitized Solar Cells. *J. Alloys Compd.* **2022**, 922, 166296. https://doi.org/10.1016/j.jallcom.2022.166296.

(5) Wang, X.; Liu, S.; Lin, S.; Qi, K.; Yan, Y.; Ma, Y. Visible Light Motivated the Photocatalytic Degradation of P-Nitrophenol by Ca2+-Doped AgInS2. *Molecules* **2024**, *29* (2), 361. https://doi.org/10.3390/molecules29020361.

(6) Yang, J.; Gu, X.; Yang, L.; Li, M.; Yan, Y.; Zhang, Y. Effect of Multi-Walled Carbon Nanotubes on the Visible Light Photocatalytic Activity of AgInS2 for Rhodamine B Degradation. *Diam. Relat. Mater.* **2024**, 141, 110682. https://doi.org/10.1016/j.diamond.2023.110682.

(7) Fu, X.; Tao, J.; He, Z.; Gao, Y.; Xia, Y.; Zhao, Z. Enhanced Photo-Reduction Performance of CuInS2 for Aqueous Cr(VI) via Facile Combining with Bi2S3: A Direct Z-Scheme Mechanism. *Journal of Materials Science: Materials in Electronics* **2022**, *33* (32), 24663–24676. https://doi.org/10.1007/s10854-022-09175-9.

(8) Zhang, J.; Zhao, Y.; Qi, K.; Liu, S. yuan. CuInS2 Quantum-Dot-Modified g-C3N4 S-Scheme Heterojunction Photocatalyst for Hydrogen Production and Tetracycline Degradation. *J. Mater. Sci. Technol.* **2024**, *172*, 145–155. https://doi.org/10.1016/j.jmst.2023.06.042.

(9) Kang, X.; Gao, T.; Lü, W.; Jiang, M.; Zhu, H.; Wang, H. Realizing Efficient Near-Infrared Emission in Cu-In-S/ZnS Quantum Dots: Aqueous Synthesis, Optical Properties and Applications. *Colloids Surf. A Physicochem. Eng. Asp.* **2024**, 701, 134966. https://doi.org/10.1016/j.colsurfa.2024.134966.

(10) Elsheikh, A. E.; Ghali, M.; Mosaad, M. M.; Sharshar, T. Studying the Effect of CuInSe2 Quantum Dot Size on the Distribution of Surface Defects Using Positron Annihilation Spectroscopy. *Mater. Sci. Semicond. Process.* **2025**, 197, 109715. https://doi.org/10.1016/j.mssp.2025.109715.

(11) Lee, H. C.; Kim, H.; Kim, K.; Lee, K.; Chung, W.; Ha, S. B.; Kim, M.; Ahn, E.; Li, S.; Ji, S.; Lee, G.; Ma, H.; Lim, S. J.; Choi, H.; Kim, J. Y.; Ahn, H.; In, S. Il; Yang, J. Unveiling Formation Pathways of Ternary I–III–VI CuInS2 Quantum Dots and Their Effect on Photoelectrochemical Hydrogen Generation. *Advanced Science* **2025**, 12, e00829. https://doi.org/10.1002/advs.202500829.

(12) Li, N.; Ma, J.; Wang, W.; Chang, Q.; Liu, L.; Hao, C.; Zhang, H.; Zhang, H.; Hu, S.; Wang, S. Dual S-Scheme MoS2/ZnIn2S4/Graphene Quantum Dots Ternary Heterojunctions for Highly Efficient Photocatalytic Hydrogen Evolution. *J. Colloid Interface Sci.* **2024**, *676*, 496–505. https://doi.org/10.1016/j.jcis.2024.07.144.

(13) Wang, H.; Jiang, H.; Wang, H.; Liu, Q.; Huo, P. NiS Cocatalyst–Modified ZnIn2S4 as Ohmic-Junction Photocatalyst for Efficient Conversion of CO2. *Energy Technology* **2022**, *10* (6), 2101158. https://doi.org/10.1002/ente.202101158.

(14) Yang, L.; Chen, Z.; Wang, X.; Jin, M. High-Stability Ti3C2-QDs/ZnIn2S4/Ti(IV) Flower-like Heterojunction for Boosted Photocatalytic Hydrogen Evolution. *Nanomaterials* **2022**, *12* (3), 542. https://doi.org/10.3390/nano12030542.

(15) Song, J.; Ma, C.; Zhang, W.; Yang, S.; Wang, S.; Lv, L.; Zhu, L.; Xia, R.; Xu, X. Tumor Cell-Targeted Zn3In2S6 and Ag-Zn-In-S Quantum Dots for Color Adjustable Luminophores. *J. Mater. Chem. B* **2016**, *4* (48), 7909–7918. https://doi.org/10.1039/c6tb02297a.

(16) Li, S.; Gong, X.; Li, Z.; Yu, M.; Chen, Y.; Yu, H.; Wang, S.; Shao, H.; Dou, M.; Cheng, Y. Interfacial Nucleation Mechanism of Water-Soluble Ag-In-S Quantum Dots at Room Temperature and Their Visible Light Catalytic Performance. *Langmuir* **2022**, *38* (15), 4692–4701. https://doi.org/10.1021/acs.langmuir.2c00236.

(17) Mir, I. A.; Bhat, M. A.; Muhammad, Z.; Rehman, S. U.; Hafeez, M.; Khan, Q.; Zhu, L. Differential and Comparative Sensing Modes of AIS and AIS@ZnS Core-Shell Quantum Dots towards Bioanalytes. *J. Alloys Compd.* **2019**, 811, 151688. https://doi.org/10.1016/j.jallcom.2019.151688.

(18) Hu, X.; Chen, T.; Xu, Y.; Wang, M.; Jiang, W.; Jiang, W. Hydrothermal Synthesis of Bright and Stable AgInS2 Quantum Dots with Tunable Visible Emission. *J. Lumin.* **2018**, *200*, 189–195. https://doi.org/10.1016/j.jlumin.2018.04.025.

(19) Chen, Y.; Chen, T.; Qin, Z.; Xie, Z.; Liang, M.; Li, Y.; Lin, J. Rapid Synthesis of AgInS2 Quantum Dots by Microwave Assisted-Hydrothermal Method and Its Application in White Light Emitting Diodes. *J. Alloys Compd.* **2023**, 930, 167389. https://doi.org/10.1016/j.jallcom.2022.167389.

(20) Korepanov, O.; Kozodaev, D.; Aleksandrova, O.; Bugrov, A.; Firsov, D.; Kirilenko, D.; Mazing, D.; Moshnikov, V.; Shomakhov, Z. Temperature- and Size-Dependent Photoluminescence of CuInS2 Quantum Dots. *Nanomaterials* **2023**, *13* (21), 2892. https://doi.org/10.3390/nano13212892.

(21) Santos, C. I. L.; Machado, W. S.; Wegner, K. D.; Gontijo, L. A. P.; Bettini, J.; Schiavon, M. A.; Reiss, P.; Aldakov, D. Hydrothermal Synthesis of Aqueous-Soluble Copper Indium Sulfide Nanocrystals and Their Use in Quantum Dot Sensitized Solar Cells. *Nanomaterials* **2020**, *10* (7), 1–14. https://doi.org/10.3390/nano10071252.

(22) Jose Varghese, R.; Parani, S.; Adeyemi, O. O.; Remya, V. R.; Sakho, E. H. M.; Maluleke, R.; Thomas, S.; Oluwafemi, O. S. Green Synthesis of Sodium Alginate Capped -CuInS2 Quantum Dots with Improved Fluorescence Properties. *J. Fluoresc.* **2020**, *30* (6), 1331–1335. https://doi.org/10.1007/s10895-020-02604-0.

(23) Ebadi, M.; Ramezani, M.; Zarghami, Z. A Facile Hydrothermal Route to the Synthesis of ZnIn2S4 Quantum Dots in the Presence of Thioglycolic Acid and Investigation Its Light Harvesting Application. *J. Clust. Sci.* **2016**, *27* (1), 341–350. https://doi.org/10.1007/s10876-015-0933-3.

(24) Zikalala, N.; Parani, S.; Tsolekile, N.; Oluwafemi, O. S. Facile Green Synthesis of ZnInS Quantum Dots: Temporal Evolution of Their Optical Properties and Cell Viability against Normal and Cancerous Cells. *J. Mater. Chem. C Mater.* **2020**, *8* (27), 9329–9336. https://doi.org/10.1039/d0tc02098b.

(25) Zikalala, N.; Parani, S.; Oluwafemi, O. S. Aqueous Synthesis of Zn-Based Ternary Core/Shell Quantum Dots with Excellent Stability and Biocompatibility against Different Cell Lines. *J. Mater. Sci.* **2022**, *57* (12), 6780–6789. https://doi.org/10.1007/s10853-022-07053-7.
